# Nuclear Perilipin 5 integrates lipid droplet lipolysis with PGC-1α/SIRT1-dependent transcriptional regulation of mitochondrial function

**DOI:** 10.1038/ncomms12723

**Published:** 2016-08-24

**Authors:** Violeta I. Gallardo-Montejano, Geetu Saxena, Christine M. Kusminski, Chaofeng Yang, John L. McAfee, Lisa Hahner, Kathleen Hoch, William Dubinsky, Vihang A. Narkar, Perry E. Bickel

**Affiliations:** 1 Division of Endocrinology, Department of Internal Medicine, The University of Texas Southwestern Medical Center, Dallas, Texas 75390, USA; 2 Center for Metabolic and Degenerative Diseases, The Brown Foundation Institute of Molecular Medicine for The Prevention Of Human Diseases, UT Health, Houston, Texas 77030, USA; 3 Touchstone Diabetes Center, Department of Internal Medicine, The University of Texas Southwestern Medical Center, Dallas, Texas 75390, USA

## Abstract

Dysfunctional cellular lipid metabolism contributes to common chronic human diseases, including type 2 diabetes, obesity, fatty liver disease and diabetic cardiomyopathy. How cells balance lipid storage and mitochondrial oxidative capacity is poorly understood. Here we identify the lipid droplet protein Perilipin 5 as a catecholamine-triggered interaction partner of PGC-1α. We report that during catecholamine-stimulated lipolysis, Perilipin 5 is phosphorylated by protein kinase A and forms transcriptional complexes with PGC-1α and SIRT1 in the nucleus. Perilipin 5 promotes PGC-1α co-activator function by disinhibiting SIRT1 deacetylase activity. We show by gain-and-loss of function studies in cells that nuclear Perilipin 5 promotes transcription of genes that mediate mitochondrial biogenesis and oxidative function. We propose that Perilipin 5 is an important molecular link that couples the coordinated catecholamine activation of the PKA pathway and of lipid droplet lipolysis with transcriptional regulation to promote efficient fatty acid catabolism and prevent mitochondrial dysfunction.

## Abstract (web summary)

Perilipin 5 has been implicated as a modulator of lipase activity and a scaffold linking lipid droplets to mitochondria. Here the authors show that PKA stimulates Peripilin 5 interaction with nuclear PGC-1a and SIRT1 to promote transcription of genes regulating mitochondrial biogenesis and respiration.

## Graphical abstract

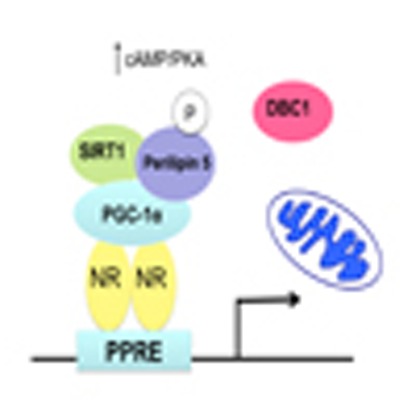

During increased energy demand, fatty acids hydrolyzed from triacylglycerol stored in cytoplasmic lipid droplets fuel energy production via mitochondrial β-oxidation and oxidative phosphorylation^1^,^2^. Excessive delivery of fatty acids to mitochondria results in incomplete fatty acid oxidation, mitochondrial inefficiency and insulin resistance^3^,^4^ that may be prevented by activation of Peroxisome proliferator-activated receptor gamma coactivator 1-alpha (PGC-1α) (ref. 4), a transcriptional regulator of mitochondrial biogenesis and function^5^,^6^,^7^,^8^. How cells sense the flux of fatty acids from lipid droplets in order to coordinately boost mitochondrial metabolic capacity is poorly understood.

Perilipin 5 is a member of the Perilipin family of lipid droplet coat proteins, which by virtue of their localization on the surface of lipid droplets regulate access of cytoplasmic lipases to the neutral lipid stored within^9^,^10^. In contrast to Perilipin 1, which is most highly expressed in white adipose tissue^11^, the major lipid storage depot in mammals, Perilipin 5 is expressed in tissues with high oxidative capacity including heart, liver, brown adipose tissue (BAT) and oxidative skeletal muscle^12^. The role of Perilipin 5 as a cytoplasmic lipid droplet protein that coordinates the actions of lipases at the lipid droplet surface and may mediate interactions between lipid droplets and mitochondria has been investigated and reviewed^12^,^13^,^14^,^15^,^16^,^17^. In addition to localizing to lipid droplets and mitochondria, pools of Perilipin 5 are present in the cytosol and endoplasmic reticulum^18^,^19^, but its functions at these sites are unknown. Consistent with its tissue distribution, Perilipin 5 gain-of-function in cells increases gene expression of enzymes involved in oxidative catabolism and promotes both triacylglycerol (TAG) storage and fatty acid oxidation^12^. In accord with these *in vitro* findings, Perilipin 5 skeletal muscle protein positively correlates with both TAG content and palmitate oxidation^16^. The increase in both TAG storage and fatty acid catabolic capacity suggests that Perilipin 5 promotes flux of fatty acid substrates into and out of the storage depot in order to facilitate rapid mobilization of energy in times of increased demand. However, the molecular mechanisms by which Perilipin 5 contributes to oxidative metabolism remain largely unknown.

To better understand how Perilipin 5 responds to metabolic conditions and whether changes in its organellar localization may influence its function, we investigated the subcellular distribution of Perilipin 5 in mouse tissues and in cultured cells under fasting and catecholamine-stimulated conditions, respectively. Surprisingly, we identified significant enrichment of Perilipin 5 in nuclear fractions under these conditions. In this work, as reported herein, we demonstrate that during catecholamine-stimulated lipolysis Perilipin 5 enriches in the nucleus, forms transcriptional complexes with PGC-1α and SIRT1, and promotes the PGC-1α gene program, thereby identifying Perilipin 5 as a previously unrecognized transcriptional co-regulator that helps match mitochondrial capacity to the lipid load.

## Results

### Perilipin 5 regulates cellular lipid metabolism

To further investigate the cell autonomous functions of Perilipin 5 in lipid metabolism, we used siRNA duplexes to knockdown its expression in MLTC-1 mouse Leydig tumour cells, which express endogenous Perilipin 5. We observed that Perilipin 5 knockdown attenuates TAG accumulation, catecholamine-stimulated glycerol release, fatty acid oxidation and ATP levels but promotes basal glycerol release (Supplementary Fig. 1). These results are consistent with the gain-of-function studies referenced above and confirm that Perilipin 5 has an important role in regulating cellular lipid metabolism.

### Triggered enrichment of Perilipin 5 in the nucleus

**Figure 1:**
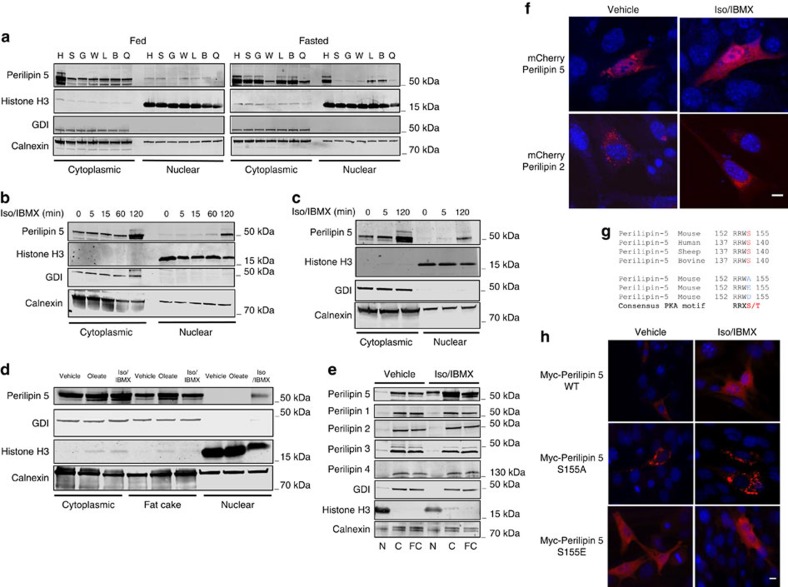
Fasting and catecholamine stimulation induce Perilipin 5 enrichment in the nucleus. (**a**) WB for Perilipin 5 in nuclear and cytoplasmic fractions prepared from heart (H), soleus (S), gastrocnemius (G), white adipose tissue (W), liver (L), BAT (B) and quadriceps (Q), of wild-type mice fed (left) or fasted (right) for 16 h. Data are representative of two independent experiments (each sample represents pooled fractions of three mice). (**b**,**c**) Time-dependent nuclear enrichment of Perilipin 5 during isoproterenol (Iso, 10 μM) and 3-isobutyl-1-methylxanthine (IBMX, 0.5 mM) treatment of C2C12 myotubes (**b**) and MLTC-1 cells (**c**). (**d**) Subcellular distribution of Perilipin 5 in nuclear, cytoplasmic and fat cake fractions after treatment of MLTC-1 cells with albumin-bound oleate (1.2 mM oleate for 16 h), vehicle or Iso/IBMX (2 h). (**e**) Subcellular distribution of Perilipin family proteins, Perilipins 1–5, in nuclear (N), cytoplasmic (C) and fat cake (FC) fractions after treatment of MLTC-1 cells with vehicle or Iso/IBMX for 2 h. (**f**) Localization of mCherry-Perilipin 5 or mCherry-Perilipin 2 in C2C12 myoblasts after treatment with vehicle or Iso/IBMX for 2 h. Nucleus stained with DAPI (blue). (**g**) Mouse Perilipin 5 PKA substrate consensus sequence is conserved among mammalian species. Amino acids identified by MS are shown in blue, and phosphoserine site is shown in red. (**h**) Immunofluorescence localization of myc-Perilipin 5 in C2C12 myoblasts transfected with wild-type myc-Perilipin 5, myc-Perilipin 5 S155A or myc-Perilipin 5 S155E after treatment with vehicle or Iso/IBMX for 2 h. Myc-Perilipin 5 wild-type and mutant proteins are shown in red, and the nucleus in blue (DAPI). Scale bar, 10 μm.

We prepared subcellular fractions from tissues of mice fed *ad libitum* or fasted for 16 h. We observed that Perilipin 5 protein increased upon fasting in whole-cell lysate (Supplementary Fig. 2a) as well in the nucleus and cytoplasm (Fig. 1a). The significant nuclear enrichment of Perilipin 5 upon fasting as compared with the fed mice is remarkable because to date no Perilipin family member has been reported to localize in the nucleus under these conditions.

To confirm and further characterize nuclear localization of Perilipin 5, we utilized two cell lines that express endogenous Perilipin 5: the C2C12 skeletal muscle cell line and the MLTC-1 cell line. We treated cells with isoproterenol (Iso) and 3-isobutyl-1-methylxanthine (IBMX) in order to activate the β-adrenergic/PKA pathway and to model a major neuro-humoral aspect of prolonged fasting. First, we found that Iso/IBMX treatment increased Perilipin 5 protein expression in both cell lines (Supplementary Fig. 2b,c). We found that in C2C12 myotubes catecholamine stimulation induced clear nuclear enrichment of Perilipin 5 by 120 min (Fig. 1b; quantification in Supplementary Fig. 2d); this enrichment appeared to reach a maximum at 120 min and was reversible starting at least 2 h after Iso/IBMX medium was replaced with regular growth medium (Supplementary Fig. 2e). Iso/IBMX treatment of MLTC-1 cells also led to nuclear enrichment of Perilipin 5 (Fig. 1c; quantification in Supplementary Fig. 3a), which is consistent with our finding of nuclear enrichment of Perilipin 5 in multiple tissues upon prolonged fasting. As with Iso/IBMX treatment, oleate loading of MLTC-1 cells resulted in increased Perilipin 5 protein expression (Supplementary Fig. 3b). However in contrast to Iso/IBMX treatment, oleate loading was not associated with nuclear enrichment of Perilipin 5 (Fig. 1d; quantification in Supplementary Fig. 3c). These data suggest that nuclear enrichment of Perilipin 5 is not due simply to increased cellular Perilipin 5 levels but rather reflects specific signalling and trafficking events.

To determine whether nuclear enrichment is a general property of the Perilipin family, we isolated nuclear, cytoplasmic and fat cake fractions from MLTC-1 cells after treatment with vehicle or Iso/IBMX for 120 min. Notably, only Perilipin 5 protein expression was increased with Iso/IBMX (Supplementary Fig. 4a), and only Perilipin 5 was enriched in the nucleus with catecholamine treatment compared with vehicle, thereby suggesting that dynamic nuclear enrichment is a unique property of Perilipin 5 within the Perilipin family (Fig. 1e; quantification in Supplementary Fig. 4b).

We used two additional independent methods to confirm catecholamine-stimulated nuclear enrichment of Perilipin 5. First, we expressed mCherry-Perilipin 5 or Perilipin 2 fusion proteins in transfected C2C12 myoblasts and assessed for red fluorescence under vehicle and Iso/IBMX-treated conditions. Consistent with our western blotting data of endogenous Perilipin 5 in C2C12 myotubes and MLTC-1 cells, the signal for mCherry-Perilipin 5 increased in the nucleus compared with basal conditions by 120 min of Iso/IBMX stimulation. On the other hand, the mCherry-Perilipin 2 signal did not enrich in the nucleus (Fig. 1f, quantification in Supplementary Fig. 5a). These data suggest that the western blotting data do not reflect antibody cross-reactivity. We confirmed by western blot (WB) that the nuclear signal for mCherry-Perilipin 5 did not originate from mCherry flourophore that had been cleaved from the mCherry-Perilipin 5 fusion protein (Supplementary Fig. 5b). Second, we examined the distribution of endogenous Perilipin 5 by immunofluorescence microscopy in MLTC-1 cells (Supplementary Fig. 6a), and found that endogenous Perilipin 5 localizes to the cytoplasm under basal conditions but is present in both the cytoplasm and the nucleus under catecholamine-stimulated conditions.

Our western blotting data indicate that nuclear enrichment of Perilipin 5 begins at least as early as 5 min after the initiation of catecholamine treatment of both C2C12 myotubes and MLTC-1 cells. This early effect suggests that at least some of the Perilipin 5 that localizes to the nucleus likely comes from existing pools. Indeed, when we repeated catecholamine treatment in the presence of cycloheximide to inhibit new protein synthesis, we observed persistent Iso/IBMX-dependent Perilipin 5 nuclear enrichment, which suggests that nuclear enrichment results at least in part from Perilipin 5 mobilization from existing depots (Supplementary Fig. 6b) as opposed to new synthesis.

To address how Perilipin 5 targets to the nucleus, we used sequence analysis software (SeqNLS and NucPred) to search for potential nuclear localization signals (NLS), but none were identified within Perilipin 5.

### Perilipin 5 nuclear localization is PKA-dependent

Perilipin-1 was initially identified as the major PKA substrate in adipocytes^11^ and recent ^32^P-orthophosphate labelling studies have shown that Perilipin 5 is phosphorylated after treatment with forskolin^15^. Using Perilipin 5 immunoprecipitation and tandem mass spectrometry (MS/MS), we identified a peptide of mouse Perilipin 5 phosphorylated on serine 155 after treatment with Iso/IBMX (Supplementary Fig. 7a). Notably serine 155 lies in a PKA consensus motif (RRXS/T) that is conserved among mammals (Fig. 1g). We confirmed that Perilipin 5 phosphorylation is PKA-dependent in cells treated with specific PKA inhibitors (PKI and RpcAMP) and Iso/IBMX or vehicle (Supplementary Fig. 7b). To determine if serine 155 phosphorylation affects the subcellular localization of Perilipin 5, we expressed in C2C12 myoblasts mutants of mouse Perilipin 5 that were either nonphosphorylatable, serine 155 to alanine (S155A), or phosphomimetic, serine 155 to glutamic acid (S155E). Notably, the S155A mutant did not leave its punctate cytoplasmic localization nor enrich in the nucleus during Iso/IBMX treatment; whereas, the S155E mutant localized to both the cytoplasm and the nucleus even in the absence of Iso/IBMX (Fig. 1h; quantification in Supplementary Fig. 8a). As was the case for mCherry-Perilipin 2, transfected wild-type Perilipin 2 did not localize in the nucleus with Iso/IBMX treatment (Supplementary Fig. 8b). In addition, we performed nuclear/cytoplasmic fractionation and WB to confirm the subcellular localization of the Perilipin 5 mutants (Supplementary Fig. 8c).

### Perilipin 5 physically interacts with PGC-1α/SIRT1 complexes

**Figure 2:**
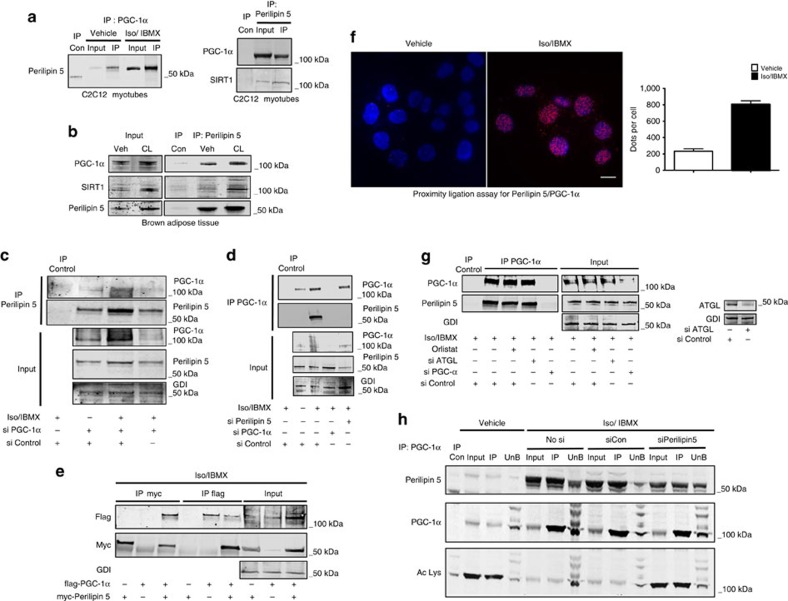
Catecholamine treatment triggers Perilipin 5 interaction with PGC-1α and SIRT1 that promotes PGC-1α deacetylation. (**a**) Immunoprecipitation (IP) of PGC-1α (left) and of Perilipin 5 (right) from nuclear extracts of C2C12 myotubes treated with vehicle or Iso/IBMX for 2 h. (**b**) IP of Perilipin 5 from BAT of wild-type mice treated with β3 adrenergic agonist CL 316,243 (CL) or vehicle. (**c**,**d**) IP of Perilipin 5 (**c**) or PGC-1α (**d**) from whole-cell lysate of brown adipocytes treated with vehicle or Iso/IBMX for 2 h. IgG control IP and siRNA duplexes for PGC-1α and/or Perilipin 5 were used as IP controls. (**e**) IP from whole-cell extract of C2C12 myoblasts transfected with myc-Perilipin 5 and flag-PGC-1α and treated with Iso/IBMX for 2 h. Non-transfected cells were used as IP control. (**f**) PLA reveals Perilipin 5 interaction with PGC-1α in C2C12 differentiating myoblasts treated with vehicle or Iso/IBMX for 2 h. Red dots indicate proximity of interaction partners within 20–40 nm (left) with quantification using Cell Profiler software (right). (**g**) IP of PGC-1α from whole-cell lysate of brown adypocytes treated with Iso/IBMX for 2 h. Cells were additionally treated with orlistat or vehicle and siATGL or siControl. IgG control and siPGC-1α were used as IP controls. (**h**) IP of PGC-1α from nuclear extracts of MLTC-1 cells transfected with siControl or siPerilipin 5. Non-immune rabbit IgG was used as the IP-negative control.

We next asked what function Perilipin 5 might have in the nucleus, and specifically whether Perilipin 5 directly regulates gene expression. We treated OP9 mouse adipocytes that stably expressed myc-Perilipin 5 (ref. 12) with Iso/IBMX or vehicle for 2 h and prepared whole-cell lysates. Immunoprecipitation of myc-Perilipin 5 from these lysates was followed by identification of interacting polypeptides by mass spectrometry (MS), which revealed an Iso/IBMX-dependent peptide sequence that corresponded to PGC-1α, thereby suggesting formation of a catecholamine-stimulated Perilipin 5/PGC-1α complex. PGC-1α expression and activity are finely tuned to reflect energy needs so that conditions of increased energy demand induce its expression^8^,^20^,^21^. Further, both isoproterenol and forskolin induce PGC-1α expression^22^,^23^. These findings from the literature prompted us to confirm the Perilipin 5/PGC-1α interaction by multiple independent methods. First, we confirmed in our hands that Iso/IBMX treatment increases PGC-1α expression (Supplementary Fig. 9a). Then we corroborated the Perilipin 5/PGC-1α interaction by reciprocal co-immunoprecipitation (co-IP) of endogenous PGC-1α and endogenous Perilipin 5 from nuclear extracts of C2C12 myotubes (Fig. 2a). We confirmed that the Perilipin 5/PGC-1α interaction occurs *in vivo* by co-IP of these complexes from intrascapular BAT isolated from mice treated with the β3-agonist compound CL 316,243 or vehicle (Fig. 2b).

Sirtuin 1 (SIRT1) is a well-known NAD^+^-dependent deacetylase and PGC-1α functional partner^24^. Under conditions of energy deficit manifested as high relative intracellular NAD^+^ concentrations, the deacetylase activity of SIRT1 increases, thereby resulting in deacetylation and consequent activation of PGC-1α as a nuclear receptor co-activator^25^,^26^. SIRT1 is detectable in the same PGC-1α protein complexes that are immunoprecipitated by Perilipin 5 antibody (Fig. 2a,b).

To confirm the specificity of WB bands observed after IP, we used siRNA duplexes for PGC-1α and Perilipin 5 and performed reciprocal co-IP using an immortalized BAT cell line that endogenously expresses both proteins (Fig. 2c,d). The immunoreactive bands detected by the PGC-1α and Perilipin 5 antibodies decreased significantly with siRNA knockdown of these proteins but not with non-targeting siContol, thereby confirming the specificity of the antibodies for PGC-1α and Perilipin 5. In addition, we overexpressed myc-Perilipin 5 and flag-PGC-1α and performed co-IP using flag and myc antibodies, which confirmed our previous co-IP results of the endogenously expressed proteins (Fig. 2e). These data make clear that our detection of Perilipin 5/PGC-1α complexes is not an artefact of antibody cross-reactivity. We also tested in cells transfected with wild-type Perilipin 5 or Perilipin 2 and PGC-1α whether Perilipin 2 also forms catecholamine-stimulated complexes with PGC-1α. In these transfected cells, immunoprecipitation of PGC-1α did not pulldown Perilipin 2 but did pulldown Perilipin 5 (Supplementary Fig. 10a).

To substantiate Perilipin 5/PGC-1α interaction by another independent method, we performed the proximity ligation assay (PLA) in C2C12 differentiating myoblasts that express both proteins endogenously. Iso/IBMX treatment induced formation of PLA signals (red dots) in the nucleus, thereby indicating the proximity of these proteins within <40 nm of each other. These data are consistent with the catecholamine-stimulated Perilipin 5/PGC-1α nuclear interaction revealed by co-IP (Fig. 2f). We used antibodies to Perilipin 2, primary antibodies only and probes only as negative controls (Supplementary Fig. 10b). We also immunoprecipitated Perilipin 5 and western blotted for PGC-1α, histone H3, Creb and actin. Only PGC-1α coimmunoprecipitated with Perilipin 5, which increases the likelihood that the Perilipin 5 interaction with PGC-1α is specific (Supplementary Fig. 11).

In that PKA phosphorylation of Perilipin 5 and PKA-driven activation of lipases occur in coordinated fashion during catecholamine stimulation, we next asked whether Perilipin 5/PGC-1α complex formation is lipolysis-dependent. We treated immortalized brown adipocytes with orlistat, a nonspecific lipase inhibitor, or siRNA duplexes for adipose triglyceride lipase (ATGL) in order to inhibit lipolysis by two independent methods. Despite lipase inhibition by either treatment, Iso/IBMX still triggered Perilipin 5/PGC-1α complex formation, which suggests that these complexes are catecholamine-dependent more than lipolysis-dependent (Fig. 2g). However, even if catecholamine-stimulated Perilipin 5/PGC-1α complex formation is able to proceed independently of lipolysis, in normal physiology these events occur simultaneously upon activation of PKA.

### Perilipin 5 influences PGC-1α acetylation status

Given that SIRT1 mediated PGC-1α deacetylation is one mechanism involved in the control of PGC-1α activity^25^,^26^,^27^, we decided to investigate whether Perilipin 5 influences PGC-1α acetylation status. We transfected siRNA duplexes targeting Perilipin 5 (siPerilipin 5) or non-targeting control siRNA duplexes (siCon) into MLTC-1 cells. After treatment with Iso/IBMX, we immunoprecipitated PGC-1α and then subjected the immunoprecipitates to western blotting with an antibody that recognizes acetylated lysine residues. We observed that PGC-1α is deacetylated after treatment with Iso/IBMX, in agreement with a previous report that forskolin, epinephrine and clenbuterol each promote PGC-1α de-acetylation^28^. We also found that Perilipin 5 knockdown is associated with reduced Iso/IBMX-dependent PGC-1α deacetylation. These data suggest that nuclear Perilipin 5 regulates PGC-1α deacetylation and may promote its co-activator function (Fig. 2h).

### Perilipin 5 activates PGC-1α target genes

**Figure 3:**
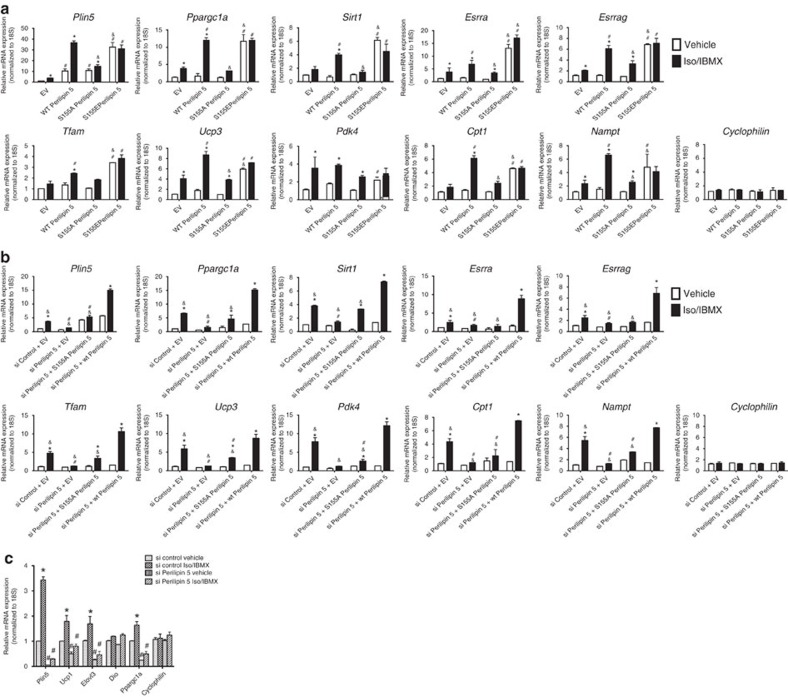
Nuclear Perilipin 5 regulates PGC-1α target genes in C2C12 myoblasts and brown adipocytes. (**a**) qPCR to detect expression of indicated genes in C2C12 myoblasts individually transfected with the following plasmids: EV, wild-type myc-Perilipin 5, S155A Perilipin 5 and S155E Perilipin 5, followed by treatment with vehicle or Iso/IBMX for 2 h. Values are mean±s.e.m. *n*=3/group. Data are representative of two independent experiments. *P<0.05 compared with vehicle and, ^#^*P*<0.05 compared with EV and ^&^*P*<0.05 compared with wild type using ANOVA followed by Tukey post-test. (**b**) qPCR to detect expression of the indicated genes in C2C12 myoblasts transfected with siControl or siPerilipin 5 duplexes with subsequent reconstitution with EV, wild-type myc-Perilipin 5 or S155A myc-Perilipin 5 followed by treatment with vehicle or Iso/IBMX for 2 h. Values are mean±s.e.m. *n*=3/group. Data are representative of two independent experiments. *P<0.05 compared with the vehicle and, ^#^*P*<0.05 compared with siControl and ^&^*P*<0.05 compared with wild type using ANOVA followed by Tukey post-test. (**c**) qPCR to detect expression of indicated genes in brown adipocytes transfected with siControl or siPerilipin 5 followed by vehicle or Iso/IBMX treatment for 2 h. For all qPCR data in Fig. 3, values are expressed as mean±s.e.m. *n*=3 biological replicates/group with two technical replicates for each biological replicate. Each biological replicate represents an independent transfection and treatment. These data were replicated in a second independent experiment. Comparative Ct method (ΔΔ Ct) was used to analyse all qPCR data. Expression of each gene was normalized to that of the 18S ribosomal subunit as endogenous control and the relative expression was calculated in comparison with reference samples as follows: (**a**) EV, vehicle (**b**) siControl + EV, vehicle, (**c**) siControl, vehicle. ANOVA, analysis of variance; EV, empty vector; qPCR, quantitative PCR.

To test whether catecholamine-triggered complex formation between Perilipin 5, PGC-1α, and SIRT1 in the nucleus affects gene expression, we overexpressed Perilipin 5 wild-type and the S155A and S155E mutants in C2C12 myoblasts, and then quantitated PGC-1α target gene expression by real-time quantitative PCR. Perilipin 5 wild-type overexpression increased PGC-1α and *SIRT1* gene expression, as well that of the PGC-1α target genes, especially after Iso/IBMX treatment, but not expression of cyclophilin (Fig. 3a), Perilipin 2, nor Perilipin 3 (Supplementary Fig. 12). The overexpression of the S155E Perilipin 5 mutant resulted in an increase of PGC-1α and its target genes even in unstimulated conditions, when compared with empty vector or wild-type Perilipin 5. When we expressed the S155A mutant, we did not observe an Iso/IBMX-stimulated increase in PGC-1α target genes (Fig. 3a). To test whether PKA-mediated phosphorylation of Perilipin 5 is required for the catecholamine-stimulated changes in gene expression we observed, we performed siRNA knockdown of Perilipin 5, as well as reconstitution with both wild-type Perilipin 5 and the S155A mutant. Consistent with our overexpression data, we observed a decrease in catecholamine-stimulated PGC-1α target gene expression when Perilipin 5 was knocked down, and that the wild type but not S155A mutant Perilipin 5 construct rescued PGC-1α target gene expression (Fig. 3b). In addition, to complement these overexpression studies, we used siRNA duplexes to knockdown Perilipin 5 in immortalized brown adipocytes and observed a decrease in the catecholamine-stimulated PGC-1α thermogenic gene program (Fig. 3c).

### Perilipin 5 complexes bind and activate the PGC-1α promoter

**Figure 4:**
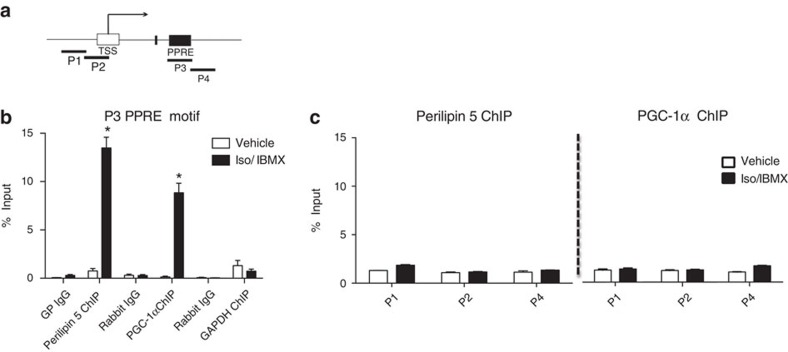
Perilipin 5 binds to PGC-1α promoter region at the PPRE motif. (**a**) P1 – 4 represent different regions of the PGC-1α promoter; P3 corresponds to the PPRE. (**b**) ChIP-qPCR assay demonstrates specific binding of Perilipin 5 and PGC-1α-containing complexes to the PGC-1α promoter region at the PPRE in C2C12 myotubes treated with Iso/IBMX or vehicle for 2 h. Control IgGs (anti-guinea pig and anti-rabbit) served as the negative controls for ChIP. (**c**) ChIP-qPCR the P1, P2 and P4 regions of the PGC-1α promoter. The results were analysed using qPCR and are shown as the percentage of input. Values are expressed as means±s.e.m. *n*=3 biological replicates/experimental group with each biological replicate representing an independent immunopreciptation for each treatment. Student *t*-test *P<0.05. Data are representative of two independent experiments (*n*=3). These data were replicated in a second independent experiment. PPRE, PPAR response element; qPCR, quantitative PCR.

Because PGC-1α co-activates its own promoter^22^, we used chromatin immunoprecipitation (ChIP) to test whether the Perilipin 5-containing complex also binds to the PGC-1α promoter region. We tested different primer sets spanning the PGC-1α promoter region (Fig. 4a), using chromatin immunoprecipitated with antibodies to Perilipin 5, to PGC-1α as a positive control, and to GAPDH as a negative control. We observed significant binding of Perilipin 5 and PGC-1α only to chromatin that contained the PPAR response element motif of the PGC-1α promoter (Fig. 4b,c).

**Figure 5:**
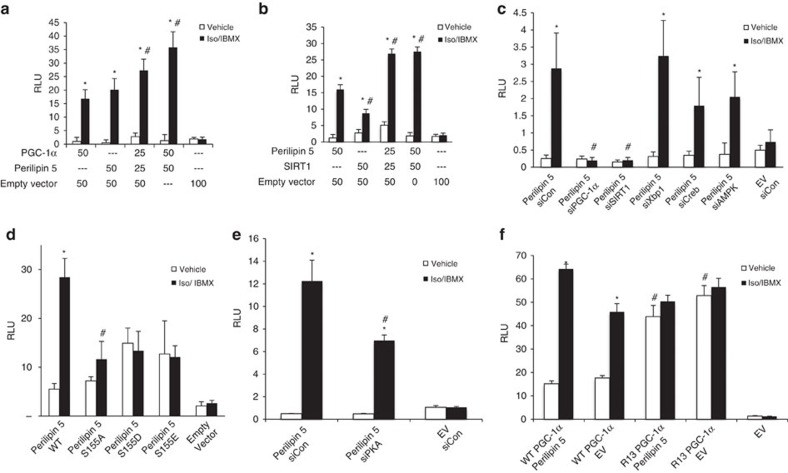
Perilipin 5 promotes catecholamine-driven PGC-1α promoter activity in a PGC-1α /SIRT1-dependent manner. C2C12 myoblasts were co-transfected with the indicated plasmids (25–100 ng each, as labelled) (**a**,**b**,**d**,**f**) or the indicated plasmids and siRNA duplexes (**c**,**e**) together with the 2 kb PGC-1α promoter luciferase plasmid. Forty-eight hours after the transfection, cells were treated with vehicle or Iso/IBMX for 2 h and luciferase activity was measured. In all experiments Renilla luciferase vector was co-transfected to normalize for transfection efficiency. Values are expressed as mean±s.e.m. *n*=6 biological replicates for each data point with each biological replicate representing an independent transfection and treatment of C2C12 myoblasts. The data shown were replicated in two additional independent experiments. *P<0.05: Iso/IBMX compared with vehicle treatment of the same transfected vectors or siRNA. ^#^*P*<0.05: the indicated transfection/treatment compared with control group. Control groups are (**a**) 50 ng PGC-1α, Iso/IBMX, (**b**) 50 ng Perilipin 5, Iso/IBMX, (**c**) Perilipin 5 + siCon, Iso/IBMX, (**d**) Perilipin 5 WT, Iso/IBMX, (**e**) Perilipin 5 + siCon, Iso/IBMX, and (**f**) PGC-1α + Perilipin 5, vehicle. Statistical analysis was done by ANOVA followed by Tukey post-test. ANOVA, analysis of variance.

To further investigate the effect of Perilipin 5 on the PGC-1α promoter, we performed luciferase reporter assays by co-transfecting C2C12 myoblasts with a previously described^22^ 2-kb PGC-1α luciferase promoter reporter construct and the expression plasmids for PGC-1α, Perilipin 5 and/or SIRT1 (Fig. 5). After treatment with vehicle or Iso/IBMX for 120 min, we observed catecholamine-dependent promoter activation by PGC-1α alone, Perilipin 5 alone and an additive effect with the combination of Perilipin 5 and PGC-1α (Fig. 5a). In addition, the combination of Perilipin 5 and SIRT1 significantly induced PGC-1α promoter activity during catecholamine treatment (Fig. 5b). To determine whether the regulation of PGC-1α promoter activity by Perilipin 5 is dependent on PGC-1α and/or SIRT1, we co-transfected Perilipin 5 and siRNA duplexes that targeted PGC-1α, SIRT1, Creb, AMPK and Xbp1. Only siSIRT1 and siPGC-1α significantly decreased Perilipin 5-mediated PGC-1α promoter activation (Fig. 5c). To determine the requirement of nuclear relocalization of Perilipin 5 for its effects on transcription, we also tested the Perilipin 5 S155A and S155E mutants for their ability to augment catecholamine-stimulated PGC-1α promoter activity. The S155A mutant had no significant effect in PGC-1α promoter activation. In contrast, the S155E mutant showed a trend to increased promoter activation under unstimulated conditions that did not reach statistical significance. Also, with the S155E mutant there was no further increase in promoter activation upon catecholamine stimulation (Fig. 5d). In addition, we transfected siRNA duplexes targeting PKA, which resulted in a significant reduction of the Perilipin 5 effect on the PGC-1α promoter (Fig. 5e). Taken together with our findings on the importance of serine 155 as a PKA substrate for Perilipin 5 localization, these data suggest that the phosphorylation of Perilipin 5 by PKA and the nuclear enrichment of Perilipin 5 are necessary for its effects on the PGC-1α promoter, and likely on other gene promoters that are co-activated by PGC-1α. That the S155E mutant under unstimulated conditions did not fully mimic the effect of wild-type Perilipin 5 in the luciferase activity under catecholamine stimulation likely reflects the fact that substitution of glutamic acid is an imperfect mimic of phosphoserine for the purposes of protein–protein interactions. As we showed in Fig. 2h, Perilipin 5 knockdown is associated with reduced Iso/IBMX-dependent PGC-1α deacetylation. To further test the effect of Perilipin 5 on PGC-1α deacetylation, we used a previously described PGC-1α acetylation deficient mutant, in which 13 lysine residues have been mutated to arginine (R13 PGC-1α) (kindly provided by Pere Puigserver)^27^. When we co-transfected wild type PGC-1α and Perilipin 5, we observed increased Iso/IBMX-stimulated PGC-1α promoter activity, as compared with wild-type PGC-1α alone. In contrast, when we transfected R13 PGC-1α, we observed increased PGC-1α promoter activity under vehicle treatment with no further increase with Iso/IBMX or Perilipin 5 co-transfection (Fig. 5f). These data suggest that the deacetylation of PGC-1α is necessary for Perilipin 5 to increase PGC-1α luciferase promoter activity.

### Perilipin 5 disinhibits SIRT1 deacetylase activity

**Figure 6:**
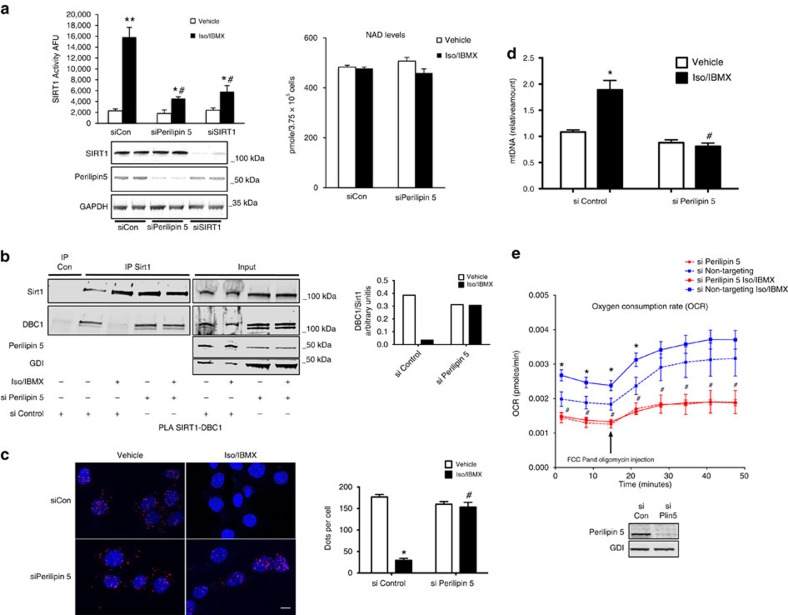
Perilipin 5 regulates catecholamine-stimulated SIRT1 deacetylation activity and mitochondrial biogenesis and respiration. (**a**) MLTC-1 cells were transfected with the indicated siRNA duplexes. After 72 h, cells were treated with Iso/IBMX or vehicle for 30 min and SIRT1 deacetylase activity was measured in the cell lysates (left). Western blotting for SIRT1, Perilipin 5 and GAPDH document siRNA specificity (bottom). Intracellular NAD^+^ levels with Iso/IBMX or siPerilipin 5 treatments (right). (**b**) Immunoprecipitation of SIRT1 followed by WB for DBC1 and SIRT1 from MLTC-1 cells transfected with the indicated siRNA duplexes. Cells were treated with vehicle or Iso/IBMX for 30 min before immunoprecipitation. Relative quantification of immunoprecipitated DBC-1 normalized for immunoprecipitated SIRT1 is shown in the histogram. (**c**) PLA for SIRT1-DBC1 complexes (red dots) in MLTC-1 cells transfected with the indicated siRNA duplexes for 48 h followed by treatment with vehicle or Iso/IBMX for 30 min (left) with quantification using Cell Profiler (right). (**d**) mtDNA content analysed by qPCR in brown adipocytes transfected with siControl or siPerilipin 5 followed by treatment with vehicle or Iso/IBMX for 2 h. (**e**) OCR in brown adipocytes transfected with siControl or siPerilipin 5 followed by treatment with vehicle or Iso/IBMX. *n*=3 biological replicates/experimental condition with each biological replicate representing an independent transfection and treatment. Values are expressed as the mean±s.e.m. These data were replicated in a second independent experiment. * *P*<0.05 compared with vehicle and ^#^*P*<0.05 compared with siControl. The data were analysed using one-way ANOVA followed by Tukey post-test. ANOVA, analysis of variance; mtDNA, mitochondrial DNA; qPCR, quantitative PCR.

Given the sum of our investigations, the simplest explanation for how Perilipin 5 exerts its function in gene regulation would be that it directly affects the deacetylase activity of SIRT1. Knockdown of Perilipin 5 is associated with reduced catecholamine-stimulated deacetylation of PGC-1α; and SIRT1, which is present in the Perilipin 5/PGC-1α complex, is well known to promote PGC-1α activity via deacetylation. We therefore tested whether Perilipin 5 regulates SIRT1 deacetylase activity by measuring vehicle and catecholamine-stimulated SIRT1 deacetylase activity in lysates of MLTC-1 cells in which Perilipin 5 or SIRT1 had been knocked down with siRNA. Knockdown of Perilipin 5 reduced Iso/IBMX-stimulated SIRT1 deacetylase activity to levels comparable to knockdown of SIRT1 itself (Fig. 6a, left). Knockdown of Perilipin 5 had no effect on NAD^+^ levels (Fig. 6a, right). These data suggest that Perilipin 5 is required for catecholamine regulation of SIRT1 deacetylase activity in a manner that does not involve changes in total cellular NAD^+^ levels.

Deleted in breast cancer 1 (DBC1) interacts with SIRT1 and inhibits its deacetylase activity^29^,^30^. Forskolin treatment leads to dissociation of the SIRT1-DBC1 complex, thereby de-repressing SIRT1 deacetylase activity^31^. On the basis of these published findings, we decided to investigate whether Perilipin 5 regulates the SIRT1-DBC1 complex. First, we assessed whether the effect of catecholamines on dissociation of the SIRT1-DBC1 complex depends on Perilipin 5. We found that knockdown of Perilipin 5 with siRNA duplexes prevented dissociation of the SIRT1-DBC1 complex in response to Iso/IBMX treatment, whereas such dissociation still occurred in cells transfected with non-targeting control siRNA duplexes (Fig. 6b). We obtained equivalent results in MLTC-1 cells co-transfected with myc-DBC1 and HA-SIRT1 (Supplementary Fig. 13), thereby reducing the possibility that our results are due to nonspecific immunoreactivity of the DBC-1 or SIRT1 antibodies. We confirmed these co-IP results with immunofluorescence microscopy using the PLA for endogenous SIRT1/DBC1 complexes (Fig. 6c). These data suggest that Perilipin 5 is a regulator of the SIRT1-DBC1 complex during catecholamine stimulation.

### Perilipin 5 regulates mitochondrial biogenesis and function

PGC-1α plays an important role in mitochondrial biogenesis and energy metablolism^5^,^6^. We next investigated whether the effects of Perilipin 5 on the *PGC-1α* gene program have functional consequences on mitochondrial biogenesis and function. We measured mitochondrial DNA in immortalized brown adipocytes (generously provided by Patrick Seale) and found that after treatment with Iso/IBMX mitochondrial DNA was increased, but that when we transfected Perilipin 5 siRNA duplexes, this increase was blunted (Fig. 6d). In addition, we measured oxygen consumption rate (OCR) in brown adipocytes that had been transfected with siControl or siPerilipin 5. Knockdown of Perilipin 5 was associated with lower basal OCR as compared with siControl. After treatment with Iso/IBMX, OCR increased in cells transfected with siControl; however, there was no Iso/IBMX associated increase in OCR in brown adipocytes treated with siPerilipin 5.

## Discussion

**Figure 7:**
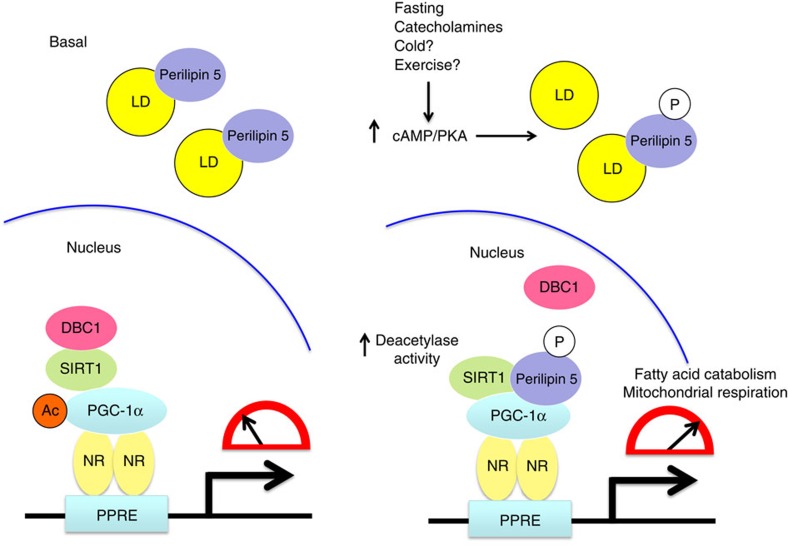
Model of the proposed mechanism for Perilipin 5 regulation of PGC-1α/SIRT1-dependent transcriptional activation. During stimulation of cells or tissues by catecholamines, fasting and perhaps other stimuli such as cold exposure and exercise, Perilipin 5 is phosphorylated by protein kinase A and enriches in the nucleus to form transcriptional complexes with PGC-1α and SIRT1. Perilipin 5 activates the deacetylase activity of SIRT1 by displacing DBC1, an inhibitor of SIRT1, thereby promoting PGC-1α co-activator activity via deacetylation. In this PGC-1α/SIRT1-dependent manner, nuclear Perilipin 5 augments expression of genes that promote mitochondrial biogenesis and function.

Perilipin 5 coats the surface of intracellular lipid droplets, where it promotes TAG storage by inhibiting lipolysis under metabolic and hormonal conditions of energy sufficiency. The exact mechanism of inhibition remains unclear, but a prevailing notion is that cytoplasmic Perilipin 5 binds either ATGL or CGI-58, an activator of ATGL lipase activity, and thereby prevents their interaction with each other^13^. This Perilipin 5 brake on unfettered lipolysis may prevent chronic overload of mitochondrial capacity to fully catabolize the released fatty acids, thereby maintaining mitochondrial function. Our data herein (Supplementary Fig. 1) and that of others^15^ suggest that in response to catecholamine treatment, Perilipin 5 promotes TAG hydrolysis. But how do cells in oxidative tissues take full advantage of the liberated free fatty acids by fully catabolizing them to produce ATP or heat and avoid the accumulation of incompletely oxidized lipid species that would damage mitochondria? In this work, we demonstrate a mechanism whereby nuclear Perilipin 5/PGC-1α actions globally reprogram lipid metabolism in response to extracellular signals. We show that acutely following catecholamine treatment Perilipin 5 is phosphorylated in a PKA-dependent manner and enriches in the nucleus, where it enters a complex with SIRT1 and PGC-1α, promotes the deacetylase activity of SIRT1 by displacing the SIRT1 inhibitor DBC-1, and thereby augments deacetylation of PGC-1α and, consequently, co-activation by PGC-1α of target genes that promote fatty acid oxidation and mitochondrial efficiency (working model, Fig. 7). Thus, whether Perilipin 5 inhibits or stimulates lipolysis and downstream fatty acid catabolism varies according to cellular energy status, hormonal milieu and its subcellular localization. These collective actions of Perilipin 5 would be expected to promote catecholamine-stimulated flux of fatty acids from lipid droplets to mitochondria. We propose that the nuclear actions of Perilipin 5 via PGC-1α activation represent an important homeostatic mechanism that matches mitochondrial oxidative capacity with the increased flux of intracellular fatty acids released from lipid droplets during catecholamine stimulation. We have shown that Perilipin 5 deficiency in brown adipocytes reduces mitochondrial DNA, reduces basal OCR, and abolishes the catecholamine-stimulated increase in OCR. We speculate that this mechanism ensures that lipolysis does not overwhelm the capacity of the mitochondria to fully oxidize fatty acids and maximizes the efficiency of ATP or heat production. Indeed, such a mismatch in flux would damage mitochondria, thereby leading to mitochondrial dysfunction, reduced metabolic flexibility and potentially insulin resistance in some tissues. Collectively, these deleterious metabolic consequences of mitochondrial overload may contribute to the pathophysiology of diseases such as obesity and type 2 diabetes^3^. This working model of Perilipin 5 function is consistent with (a) the tissue toxicity that arises in global Perilipin 5 knockout mice (see below) and (b) the adaptive storage of fatty acids as triacylglycerol in lipid droplets, such as occurs with endurance training of muscle in humans, which is associated with increased Perilipin 5 gene expression and protein levels.

Our data do not reveal the mechanism by which Perilipin 5 targets to the nucleus. Perhaps as has been shown in osteoblasts for β-catenin^32^, which also lacks a nuclear localization sequence^33^, PKA phosphorylation generates a signal that directs Perilipin 5 to the nucleus. Alternatively, Perilipin 5 may contain a cryptic NLS. These issues remain for further investigation.

The role of Perilipin 5 in regulating SIRT1 activity must be considered in the context of other signalling pathways that also promote SIRT1 deacetylase activity. The Puigserver laboratory has identified a conserved serine (S434) in the catalytic domain of SIRT1 that is phosphorylated upon activation of the cAMP/PKA pathway by exposure either to catecholamines or to cold^28^ and is required for SIRT1-dependent augmentation of gene expression and fatty acid oxidation under those conditions. Notably, activation of SIRT1 neither by cAMP/PKA-dependent phosphorylation nor by its interaction with Perilipin 5 as we report is associated with increased cellular NAD^+^. These modes of NAD^+^-independent SIRT1 regulation contrast with AMP-kinase (AMPK) driven activation of SIRT1, which is mediated by increased production of NAD^+^ (refs 34, 35). How these distinct modes of regulation may interact with each other remains for future study, but it is likely that PKA-mediated phosphorylation of SIRT1 results in rapid augmentation of SIRT1 de-acetylase activity that may be maintained through AMPK-driven changes in NAD^+28^. Our data suggest that Perilipin 5 exerts its effect on SIRT1 activity early following catecholamine stimulation and on gene expression within a few hours.

Our results are consistent with several previous reports of Perilipin 5 in rodent models and in human subjects. First, acute overexpression of Perilipin 5 via adenoviral delivery into mouse skeletal muscle increases intramyocellular lipid levels without impairing insulin sensitivity and increases gene expression that favours fatty acid oxidation and mitochondrial function^36^. Second, endurance trained humans have increased skeletal muscle (vastus lateralis) Perilipin-5 protein compared with lean sedentary insulin-sensitive humans^37^. In a third study, Perilipin 5 expression was upregulated in skeletal muscle of mice that specifically overexpressed PGC-1α in muscle, as well as in skeletal muscle of exercise-trained humans^38^. Notably, Perilipin 5 expression in skeletal muscle positively correlated with insulin sensitivity in those exercise-trained subjects^38^. Collectively, these studies suggest that TAG accumulation mediated by Perilipin 5 is an adaptive mechanism to buffer lipid loads and to permit healthy intracellular lipid flux.

Against the backdrop of recent data from others, our results suggest that certain aspects of Perilipin 5 may be cell-type specific. Perilipin 5 overexpression in mouse models positively correlated with increased tissue steatosis in heart^39^,^40^, skeletal muscle^36^,^41^ and liver^42^, which is consistent with our cell culture data. However, in the heart specifically, indices of mitochondrial function, gene expression related to oxidative metabolism (PPARα, PGC-1α and related genes), and fatty acid oxidation negatively correlated with Perilipin 5 expression^39^,^40^. Such differences may relate to the intrinsic capacity of the healthy adult heart to handle high rates of TAG turnover and fatty acid flux independent of Perilipin 5, a feature that dramatically changes with cardiac hypertrophy. Whether myocardial Perilipin 5 actions are adaptive in cardiac hypertrophy, for example in the context of pressure overload, remains to be determined. It is notable that PGC-1α expression positively correlates with that of TCA cycle enzymes^4^ that would enhance anaplerosis, an essential process that becomes defective in the failing heart due to suppression of malic enzyme expression^43^. Perhaps enhancement of Perilipin 5 expression in the failing heart would activate PGC-1α and improve anaplerotic replenishment of TCA cycle intermediates.

Nevertheless, in concordance with our data, whole-body Perilipin 5 knockout mouse models have shown reduced triacylglycerol content and lipid droplets in skeletal muscle^44^, liver^45^ and heart^46^. This consistent phenotype of lipid droplet depletion was associated with adverse tissue-specific sequelae, as predicted by our working model. In skeletal muscle, whole-body Perilipin 5 deficiency was associated with increased ceramide accumulation and insulin resistance^44^. In liver, whole-body Perilipin 5 deficiency was associated with increased hepatocellular non-esterified fatty acids with PPARα activation and with increased markers of ER stress, inflammation, and tissue injury (elevated transaminases)^45^. Mice with whole-body Perilipin 5 deficiency developed age-related cardiomyopathy associated with increased fatty acid oxidation^46^.

The published Perilipin 5 mouse knockout models reflect chronic and potentially developmental effects on whole-body physiology with the potential for activation of compensatory metabolic responses that may complicate the determination of the specific functions of Perilipin 5. For example, inability to sequester fatty acids in lipid droplets as triacylglycerol may lead to increased production of PPAR ligands that compensate for the loss of Perilipin 5-dependent PGC-1α activation. In contrast, our data reflect the cell autonomous effects of acute/subacute changes in Perilipin 5 expression on cellular gene expression and lipid metabolism. The relative importance of Perilipin 5 function in different organelles (lipid droplet, mitochondria and nucleus) likely is specific to the tissue being considered and to the metabolic context (fasting versus feeding, thermoneutrality versus cold, rest versus exercise). The function of Perilipin 5 as a transcriptional regulator likely has less to do with establishing the tonic level of oxidative gene expression and mitochondrial capacity than with matching gene expression and mitochondrial capacity to changing nutrient and hormonal conditions. In this regard, the dual actions of Perilipin 5 resemble those of lipin-1 in sensing and coordinating TAG metabolism^47^.

In sum, our work makes clear that Perilipin 5 functions extend beyond its role at the lipid droplet surface in regulating lipid storage. As we report for the first time herein, Perilipin 5 also functions in the transcriptional regulation of mitochondrial respiration via nuclear translocation and direct interaction of Perilipin 5 with the PGC-1α mitochondrial gene program. The transcriptional effects of nuclear Perilipin 5 on metabolism may not be entirely cell autonomous, as suggested by the recent report that Perilipin 5 overexpression in skeletal muscle is associated with increased muscle gene expression and serum levels of FGF21 (ref. 41), a PPARα target gene^48^. Future studies will determine whether nuclear Perilipin 5 regulates deacetylation states of known SIRT1 targets beyond PGC-1α, such as p53, FOXO, NFκB, LXR, CRTC2 and SREBP-1 (ref. 49) This last question is significant given the known and proposed roles of SIRT1 as an integrator of metabolic signalling relevant to cancer biology, aging, inflammation, thermogenesis and exercise, in addition to energy balance.

## Methods

### Mouse studies

For mouse experiments, we utilized adult 12-weeks-old female C57BL/6 mice. For fasting studies, mice were without access to food for 16 h, and fed mice were provided *ad libitum* access to standard rodent chow. For CL 316,243 experiments, we injected fed mice with CL 316,243 (1 μg g^−1^) or saline. Two hours after CL 316,243 injection we harvested BAT. All animal experiments were approved for the Animal Welfare Committee of University of Texas-Health Sciences Center at Houston or University of Texas Southwestern Medical Center Institutional Animal Care and Use Committee.

### Reagents

Isoproterenol (cat # I6504), IBMX (cat # I5879), Palmitate (cat # P0500-100G), Rp-Adenosine 3′,5′-cylic monophophorothioate triethylammonium salt (cat # A165-1MG), cycloheximide (cat # C1988) and oleic acid (cat # O1008) were from Sigma (St. Louis, MO, USA). PKA inhibitors PKI, 14-22 Amide, cell permeable myristoylated was from Calbiochem (cat # 476485).

Oleate complexed with albumin 5.5:1 molar ratio for cell culture treatments was prepared as previously described^50^ as follows: oleic acid was solubilized with 1 M sodium hydroxide at 60 °C, then the sodium-oleate solution was bound to fatty acid-free bovine serum albumin (Fitzgerald cat # 30-AB79) at a molar ratio of 5.5:1.

DMEM, MEM-a, RPMI1640, fetal bovine serum (FBS) and penicillin/streptomycin/L-glutamine, Lipofectamine 2000 were from Invitrogen Corp. (Carlsbad, CA, USA).

Dynabeads M280 Sheep anti-Mouse (cat # 11201D) and Sheep anti-Rabbit (cat # 2016D6) were from Invitrogen. Protein A magnetic beads were from Thermo Scientific (cat # 21348).

### Antibodies

Antibodies corresponding to COOH and NH_2_—terminal of Perilipin 5 have been described previously (dilution 1:500 for WB and 3 μg for IP)^12^. Perilipin 5 (cat # GP31, dilution 1:1,000 for WB, IF and PLA and 3 μl for IP), Perilipin 1 (cat # GP29, dilution 1:1,000 for WB), Perilipin 2 (cat # GP40, dilution 1:1,000 for WB, IF and PLA) and Perilipin 4 (cat # GP34, dilution 1:1,000 for WB) were purchased from Progen. Perilipin 3 (cat # 20R-2602, dilution 1:1,000 for WB) was from Fitzgerald. PGC-1α antibodies were from EMD Millipore (cat # ST1202 dilution 1:250 for WB,1:500 for PLA and ST1204 2-3 μg for IP ) and Santa Cruz Biotechnology (cat # sc-13067 for Fig. 2i, dilution 1:1,000 for WB and 2 μg for IP ). Antibodies for SIRT1 (cat # 07-131, dilution 1:1,000 for WB and 3 μg for IP) were from Millipore. Antibodies for acetyl-lysine (cat # 9441, dilution 1:1,000 for WB), histone H3 (cat # 9715, dilution 1:1,000 for WB), phospho PKA substrate (cat # 9624, dilution 1:1,000), GAPDH (cat # 2118, dilution 1:1,000), HA (cat # 3724, dilution 1:1,000), DBC1 (cat # 5857 dilution 1:1,000) Creb (cat #9197 dilution 1:1,000) and ATGL (cat # 2138, dilution 1:1,000) were purchased from Cell Signaling. Calnexin was from Santa Cruz Biotechnology (cat # 6465, dilution 1:500), DBC1 antibody (cat # A303-942A, dilution 1:500) from Bethyl Labs was used for PLA. Anti-myc (9E 10) was deposited to the DSHB by Bishop, J. Michael (DSHB Hybridoma Product 9E 10, dilution 2 μg for IP, 1:1,000 for WB). GDI antibody was previously described (dilution 1:2,000)^51^.

### Plasmids

The myc-Perilipin 5 expression plasmid was previously described^12^.

For mCherry plasmids: mCherry was cloned from pmCherry-N1 vector (Clontech) and inserted at the N-terminal of either Perilipin 5 or Perilipin 2 sequence under TRE promoter control. For experiments C2C12 myoblasts were co-transfected with the mCherry Perilipin-5 or mCherry Perilipin 2 plus the pTet-On Advanced vector after 24 h cells were treated with 100 ng ml^−1^ of Doxycycline in the culture medium for protein expression induction after 24 h cells were fixed and analysed using a Leica TCS SP5 confocal fluorescent microscope.

To generate the myc-DBC1 construct, we used pCMV-SPORT6_DBC1 as a template (Open Biosystems, Cat. MMM1013-202763843). We cloned myc-DBC1 into pcDNA3.1/V5-His-Topo vector at KpnI and NotI sites.

PGC-1 alpha promoter 2 kb luciferase was a gift from Bruce Spiegelman (Addgene plasmid # 8887 (ref. 22)). pCruzHA SIRT1 was a gift from Toren Finkel (Addgene plasmid # 10962 (ref. 24)). pCMX-PGC-1α was a kind gift from Dr Ron M. Evans. Wild-type PGC-1α and R13 PGC-1α were a kind gift from Pere Puigserver Lab. Plasmids for empty pGL3-basic and renilla pRL-TK were purchased from Promega.

### Cell culture

C2C12 myoblasts and mouse Leydig tumour cells (MLTC-1) were obtained from ATCC (Manassas, VA).

MLTC-1 cells were maintained in RPMI 1640 medium and 10% FBS at 37 °C and 5% CO_2_. C2C12 myoblasts were maintained in DMEM, 10% FBS at 37 °C and 5% CO_2_. For differentiation, when C2C12 myoblasts reached 70% of confluence regular media was replaced with DMEM and 2% horse serum, cells were harvested 7 days after initiation of differentiation. We produced C2C12 differentiating myoblasts by replacing regular media with differentiation media (DMEM and 2% horse serum) plus 0.2 mM oleate and 50 μM Wy-14643 24 h before the experiment. Brown adipose cell line was a kind gift from Patrick Seale (U.Penn). Cells were maintained in DMEM/F12 and 10% FBS. For differentiation growth medium was supplemented with insulin (20 nM), dexamethasone (1 uM), IBMX (500 uM) and Triiodothyronine (T3- 1 nM) for 2 days. From days 3–8 cells were in growth medium with insulin (20 nM) and+T3 (1 nM) and cells were harvested at day 7-8.

The OP9 myc-Perilipin-5 stable cell line^12^ was maintained and differentiated as follows: OP9 cells were grown in OP9 medium (MEM-α and 20% FBS) and maintained at 90–100% confluence. For differentiation cells were grown to confluence and then cultured for 2 additional days in culture medium and then cultured 4 additional days using serum replacement medium: MEM-α with 15% KnockOut SR (Gibco, catalogue no. 10828-028)

### Cell fractionation

Nuclear and cytoplasmic fractions from mouse tissues and cell lines were prepared with the Nuclear Complex Co-IP kit (Active Motif cat# 54001; Carlsbad, CA) following the manufacturer's instructions. ‘Fat cake' fraction was obtained by cytoplasmic fraction centrifugation (12,000*g* for 20 min at 4 °C) followed by collection by aspiration and resuspension in SDS sample buffer.

### Immunoprecipitation (IP)

For IP from cell fractions, we incubated nuclear and cytosolic proteins (500 μg) with 2 μg of indicated primary antibodies or non-immune***** IgG of the same species as controls in 200 μl of IP buffer (20mM HEPES pH 7.9, 0.15 M NaCl, 1 mM EDTA, 1 mM EGTA, 1 mM DTT, 1 mM PMSF and 1 × protease inhibitor cocktail) for 4 h followed by incubation for 2 h with 50 μl of washed Dynabeads (Life Technologies, Grand Island, NY) coated with the appropriate secondary antibody. After three 10 min washes with IP buffer, we resuspended the beads in SDS sample buffer and boiled for 5 min at 95 °C to elute the immunoprecitated proteins.

For IP from whole-cell lysate, we washed cells three times with cold PBS and lysed the cells in HEPES, pH 7.4, 150 mM NaCl, 2 mM EDTA, 1%Triton X-100 and 0.5% cholate with Protease Inhibitor (Roche) and Halt phosphatase inhibitor cocktail (Thermo cat # 78426). We incubated 500 μg of each cleared supernatant with Dynabeads pre-coupled with 2–3 μg of indicated primary antibody. After for 4 h incubation we washed the beads three times with cold PBS and 0.1% bovine serum albumin (BSA) and then resuspended Dynabeads in SDS sample buffer and boiled for 5 min at 95 °C. All IP incubation steps were performed at 4 °C. IP samples were loaded on 10%, 4–20% or Any/kD Criterion TGX SDS-polyacrylamide gel electrophoresis gels (BioRad, Hercules, CA). For MS, gels were stained with Coomassie blue.

### Immunoblots

Cell lysates, subcellular fractions or immunoprecipites were resolved by SDS-polyacrylamide gel electrophoresis using 10%, 4–20% or Any/kD Criterion TGX gels (BioRad, Hercules, CA) and transferred to nitrocellulose (0.2 μm) by wet transfer. Membranes were then stained with Ponceau S and preblocked in PBS-Tween-20 with 5% nonfat dry milk followed by incubation with the indicated primary and fluorescent secondary antibodies in PBS-Tween-20 with 3% fraction V BSA. Immunoblotted proteins were visualized using the Li-cor Odyssey CLx Imaging System (Li-cor Biosciences, Lincoln, NE).

Uncropped immunoblots for the most important experiments are provided in Supplementary Fig. 14.

### TAG measurements

Triacylglycerol (TAG) accumulation assay was performed in MLTC-1 cells treated with 1 mM oleate or vehicle for 16 h using organic extraction in a 96-well format as described previously^52^.

### Glycerol release assay

We treated MLTC-1 cells with Iso/IBMX using serum-free culture medium containing 0.5% fatty acid-free BSA for 2 h. Glycerol in the culture medium was measured using the Glycerol Cell-Based Assay Kit (cat# 10011725; Cayman Chemical Company, Ann Arbor, MI).

### Palmitate oxidation assays

MLTC-1 cells were plated in equivalent numbers in T25 flasks. Twenty-four hours later, the cells were given fresh growth medium containing [1-14C] palmitate (American Radiolabeled Chemicals Inc., St Louis, Missouri, USA), and #1 Whatman filter paper was suspended within each flask. The flasks were sealed, and 24 h later the cells were lysed with 6 N hydrochloric acid. The 14CO_2_ collected overnight on the Whatman paper was liberated by alkalization with 2 N sodium hydroxide, and was quantified by scintillation counting.

### ATP Measurements

Intracellular ATP levels were determined using ATP Bioluminescence Assay Kit HS 2 (Roche, Basel, Switzerland). MLTC-1 cells were grown on a 24-well plate and transfected with indicated siRNA. Two days after transfection, cells were treated with vehicle or Iso/IBMX for the indicated times, trypsinized, spun down and resuspended in the dilution buffer. Equal amounts of the cell suspension and lysis buffer were mixed gently and incubated for 5 min. Aliquots were transferred into 96-well microplates with luciferase reagent. ATP was measured immediately by using a Leica Infinite M1000 Plate Reader. The ATP standard curve was prepared with ATP provided with the kit.

### Real-time PCR

Total RNA was extracted from cells using RNeasy Mini Kit (Qiagen Cat No. 74104). We reversed transcribed using Iscript cDNA synthesis kit (Bio-Rad). Quantitative real-time PCR from synthesized cDNA was performed using a ViiA7 Sequence Detection System (Applied Biosystems, Foster City, CA) using Power SYBR Green PCR Master Mix (Applied Biosystems cat # 4367659). We use ViiA7 RUO software to perform Comparative Ct experiments (ΔΔCt) to determine the relative target quantity in samples. With the comparative Ct method, the ViiA7 software measures amplification of the target and the endogenous control (18S and cyclophilin) in samples and in a reference sample. Measurements are normalized using the endogenous control. The software determines the relative quantity of target in each sample by comparing normalized Ct (ΔCt) in each sample to normalized Ct (ΔCt) in the reference sample.

Primers were designed using Primer Express 3.0 (Applied Biosystems). Primers used in quantitative PCR are listed in Supplementary Table 1.

### Reporter gene assays

C2C12 myoblasts were plated on a 96-well plate and we co-transfected indicated amounts of relevant expression plasmids with 100 ng of PGC-1α 2-kb luciferase promoter plasmid (Addgene plasmid # 8887 (ref. 22)). To normalize for transfection efficiency 20 ng of pRL-TK reporter plasmid was added to each transfection. As required, we used the relevant empty vector to ensure that each transfection received the equivalent amount of total DNA. Forty-eight hours after transfection cells were treated with vehicle or Iso/IBMX for 120 min. We measured firefly luciferase activity and normalized to Renilla luciferase expression using Dual-Glo luciferase reporter assay system (cat # E2940; Promega, Madison, WI). For siRNA experiments the indicated siRNA duplexes were transfected 24 h before plasmid PGC-1α 2kb-luciferase promoter and expression plasmid transfection.

### *In situ* Proximity Ligation Assay (PLA)

We utilized the DuoLinkTM *in situ* PLA (Olink Bioscience, Uppsala, Sweden) according to the manufacturer's protocol. Briefly, C2C12 differentiating myoblasts were used, cells were treated with Iso/IBMX or vehicle for 2 h followed by fixation with 4% paraformaldehyde for 15 min and permeabilization in ice-cold 100% methanol for 15 min. Fixed cells were blocked for 30 min followed by incubation with the indicated primary antibodies for 2 h. Cells incubated with primary antibodies only or probes only (guinea-pig and rabbit) were used as the assay controls. Cells were then incubated with oligonucleotide-conjugated PLA probes for 60 min followed by ligation of PLA probes for 30 min and the amplification of signal for 100 min in a humidified chamber according to the manufacturer's instructions. The slides were visualized using a Carl Zeiss fluorescent microscope or Leica confocal microscope.

### Small interfering RNA (siRNA)

C2C12 myoblasts and MLTC-1 cells were grown to 50% confluence in 10 cm^2^ plates and transfected with indicated siRNA duplexes using DharmaFECT transfection reagent (Dharmacon; T-2001-01). The final siRNA concentration was 50 nM. Cells were harvested after 48 h for RNA extraction and after 72 h for subcellular fractionation and protein extraction. The siRNA was purchased from Dharmacon: Perilipin-5 (cat # J-055756-09), Non-Targeting (cat # D-001810-01-20), PGC-1α (cat # L-040773-01), SIRT1 (cat # L049440-00), Creb (cat # L-040959-01), AMPK (L-041035-00), PKA (L-047080-00) and ATGL (L-040220-01) Xbp1 siRNA was a kind gift of Dr Phillip Scherer.

### Chromatin immunoprecipitation (ChIP) assay

For ChIP assay, we used ChIP-IT assay kit (Active Motif, Carlsbad, CA, USA) according to the manufacturer's instructions. Briefly, C2C12 myotubes were crosslinked with PBS containing 1% formaldehyde for 10 min, reaction was stopped using glycine and washed three times with ice-cold PBS. Cells were then harvested, homogenized and the crosslinked protein–DNA complexes were immunoprecipitated with antibodies for Perilipin 5, PGC-1α and GAPDH. Non-immune IgG controls for the corresponding species were used as negative controls. The protein–DNA complexes were then separated by protein G-coated magnetic beads, treated with protease, and then analysed by real-time PCR with reverse transcription. Primers used are listed in Supplementary Table 2.

### Mass spectrometry

MS analysis was performed in the Proteomics Core Facility at University of Texas Health Science Center at Houston. MS/MS analysis was performed on an AppliedBiosystems QStar XL LC/MS/MS mass spectrometer equipped with an LC Packings HPLC for capillary chromatography.

The HPLC is coupled to the mass spectrometer by a nanospray ESI head for direct analysis of the eluate. Protein bands were excised from Coomasie-stained gels and subjected to in gel trypsin digestion. Peptide extracts were separated by HPLC on a C18 reverse phase capillary column (Vydac218MS3.07510) developed with a 2–50%acetonitrile gradient in 0.1% formic acid, 0.005%TFA over 30 min at a flow rate of 200 nl min^−1^. The QSTAR was operated in Information Dependent Acquisition mode using a 1 s survey scan followed by two consecutive 3 s product ion scans of 2+, 3+ and 4+ parent ions (*m*/*z* 350–1,500). Identification was performed on MASCOT with an MS and MS/MS mass tolerance of 0.15 Da.

### Site-directed mutagenesis

To generate mutants (S155A, S155E, S155D) we used as template Perilipin 5 expression construct with NH2 terminus myc epitope that was previously described^8^. We use QuickChange Lightning Site-Directed Mutagenesis Kit (Agilent Cat # 210518) using the manufacturer's protocol. Primers are listed in Supplementary Table 3.

### Immunofluorescence

C2C12 myoblasts were plated on coverslips in a 12-well tissue culture dish and transfected with the indicated constructs and/or treated as indicated. Cells were washed with PBS three times and then fixed in 3% paraformaldehyde for 20 min and permeabilization was performed in ice-cold 100% methanol for 10 min, then cells were blocked with 1 × PBS, 3% BSA and 0.01% Saponin for 1 h at room temperature, coverslips were incubated with primary antibody in 1 × PBS, 1% BSA, 0.01% Saponin for 1 h at room temperature, washed three times with PBS and incubated with secondary antibodies for 30 min in antibody dilution buffer, then washed three times with PBS and mounted with Prolong Gold anti fade reagent with or without DAPI (Invitrogen Cat # P36930 and P36931).

### Cellular SIRT1 activity Assay

MLTC-1 cells were plated in six-well tissue culture dishes and treated with the indicated siRNA duplexes; after 72 h cells were treated with vehicle or Iso/IBMX for 30 min, and the SIRT1 activity assay was performed on the cell lysates by fluorometric assay (Enzo Life Science cat # BML-AK555-0001) using a method previously used^31^ as follows: cells were extracted using 20 mM Tris-HCl, pH 8.0, 100 mM NaCl, 1 mM EDTA and 0.5% Nonidet P-40 supplemented with 5 mM NaF, 50 mM 2-glycerophosphate, 1 mM Na_3_VO_4_, and a protease inhibitor mixture (Roche Applied Science). Protein was quantified and equivalent amounts of protein were aliquoted into sample tubes. Sample volumes were equalized with deacetylase buffer (50 mM Tris-HCl, pH 8, 137 mM NaCl, 2.7 mM KCl, 1 mM MgCl_2_ and 1 mg ml^−1^ BSA). Samples were incubated for 10 min at 30 °C to allow for NAD^+^degradation and incubated for 10 additional min with 2 μM DTT. Finally, 30 μg of protein of each sample were transferred to six wells of a 96-well plate, and a solution of deacetylase buffer containing 100 μM substrate and 5 μM trichostatin A was added to the wells. Half the wells included 100 μM NAD^+^. The reaction proceeded for 2 h at room temperature, and then the developer, prepared according to the manufacturer's recommendations, was added for 1 h. Finally, the fluorescence was read with excitation of 360 nm and emission at 460 nm. SIRT1 activity was calculated as NAD^+^-dependent fluorescence.

Cellular NAD^+^ levels were measured using NAD^+^/NADH Quantitation Kit Sigma (cat # MAK037) following the manufacturer's instructions.

### Mitochondrial experiments

Brown adipocytes were growth and differentiated as described above in XFp cell culture miniplates (Seahorse bioscience) and OCR measurements were performed using XFp Extracellular Flux Analyzer with the XFp cell energy phenotype stress kit (Seahorse Bioscience, MA) following the manufacturers' protocols.

### Mitochondrial DNA quantification

For mitochondrial DNA quantification brown adipocytes were transfected with siControl or siPerilipin 5, and 72 h after transfection were treated with vehicle or Iso/IBMX for 2 h. Total DNA was extracted using QIAmp DNA kit (Qiagen Cat # 51304) following the manufacturers' instructions. Mitochondrial DNA was amplified using primers specific for the mitochondrial cytochrome c oxidase subunit 2 (COX2) gene and normalized to genomic DNA by amplification of the ribosomal protein s18 (rps18) nuclear gene using quantitative PCR. Primers used were previously described for this method^53^ and are listed in Supplementary Table 4.

### Nuclear localization signal (NLS) prediction

We used SeqNLS^54^ and NucPred^55^ for NLS prediction of mouse Perilipin 5 protein sequence (Uniprot protein identifier Q8BVZ1).

### Statistical analyses

Results were analysed using either Student's *t*-test or one-way analysis of variance with subsequent *post hoc* Tukey's pairwise analysis using GraphPad Prism version 6.00, San Diego, CA, USA. Data are presented as means±s.e.m. with statistically significant differences as *P* values <0.05. 

### Data availability

The data that support the findings of this study are available from the corresponding author upon request.

## Additional information

**How to cite this article:** Gallardo-Montejano, V. I. *et al*. Nuclear Perilipin 5 integrates lipid droplet lipolysis with PGC-1α/SIRT1-dependent transcriptional regulation of mitochondrial function. *Nat. Commun.* 7:12723 doi: 10.1038/ncomms12723 (2016).

## Supplementary Material

Supplementary Information Supplementary Figures 1-14, Supplementary Table 1-4
