# Supplementary material for: Nuclear Perilipin 5 integrates lipid droplet lipolysis with PGC-1α/SIRT1-dependent transcriptional regulation of mitochondrial function
Source: Nat Commun. 2016 Aug 24;7:12723. doi: 10.1038/ncomms12723 (PMC4999519; doi:10.1038/ncomms12723)
Supplement: Supplementary Information — Supplementary Figures 1-14, Supplementary Table 1-4 [file ncomms12723-s1.pdf]

Supplementary Figure 1

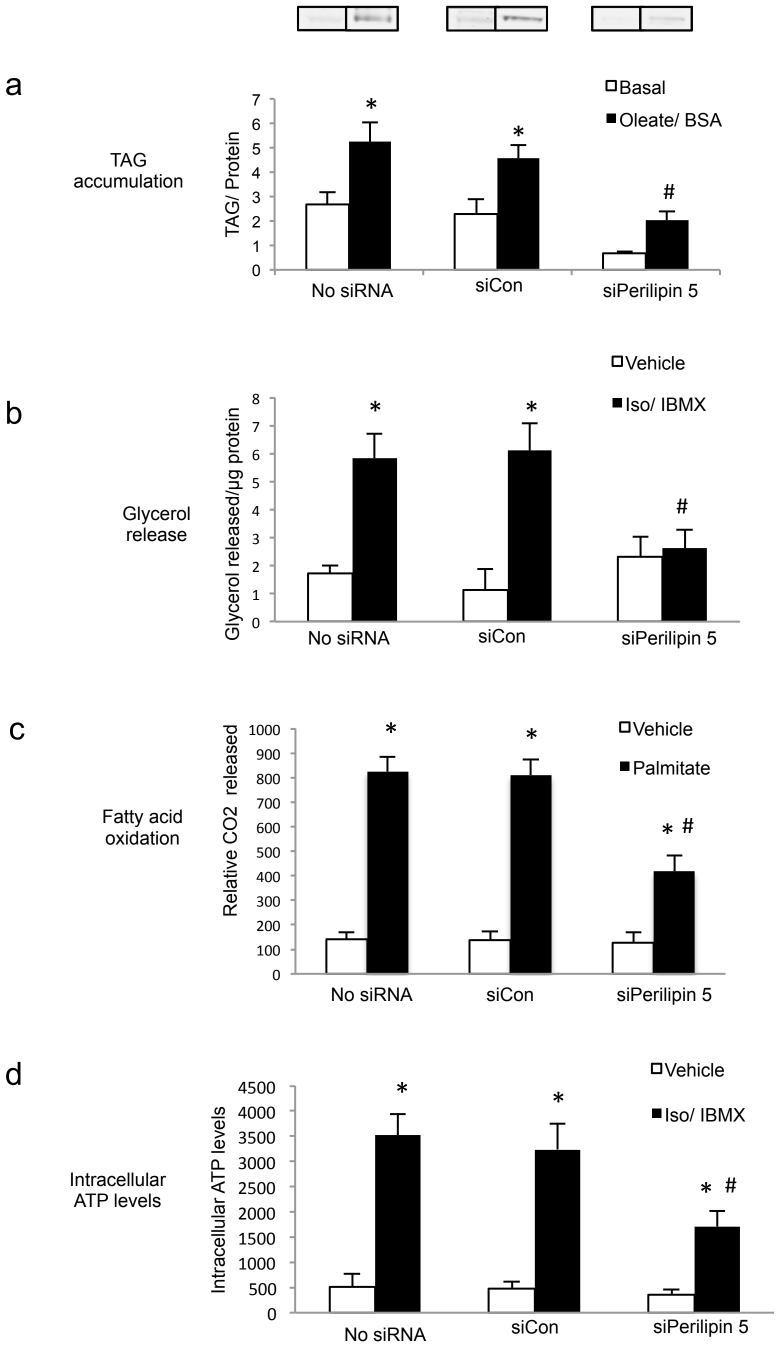

Supplementary Figure 1. Effect of Perilipin 5 deficiency on TAG accumulation, glycerol release, palmitate oxidation and ATP levels

a, TAG-to-protein ratios in MLTC-1 cells untransfected, transfected with siControl (siCon), or transfected with siPerilipin 5 followed by treatment with 1.2 mM oleate treatment for 16 h. **b,** Glycerol released/µg of protein in MLTC-1 cells transfected or not as indicated, followed by treatment with Iso/ IBMX for 2. **c,** Relative CO_2 released in MLTC-1 cells transfected or not as indicated, followed by treatment with 0.25 mM palmitate. **d,** Cellular ATP levels in MLTC-1 cells transfected or not as indicated, followed by treatment with Iso/IBMX for 5 or 120 min. Values are expressed as mean ± s.e.m n= 3. * $p < 0.05$ compared to Vehicle for the same transfection conditions; $^{\#}$ $p < 0.05$ compared to siCon, Iso/IBMX.

Supplementary Figure 2

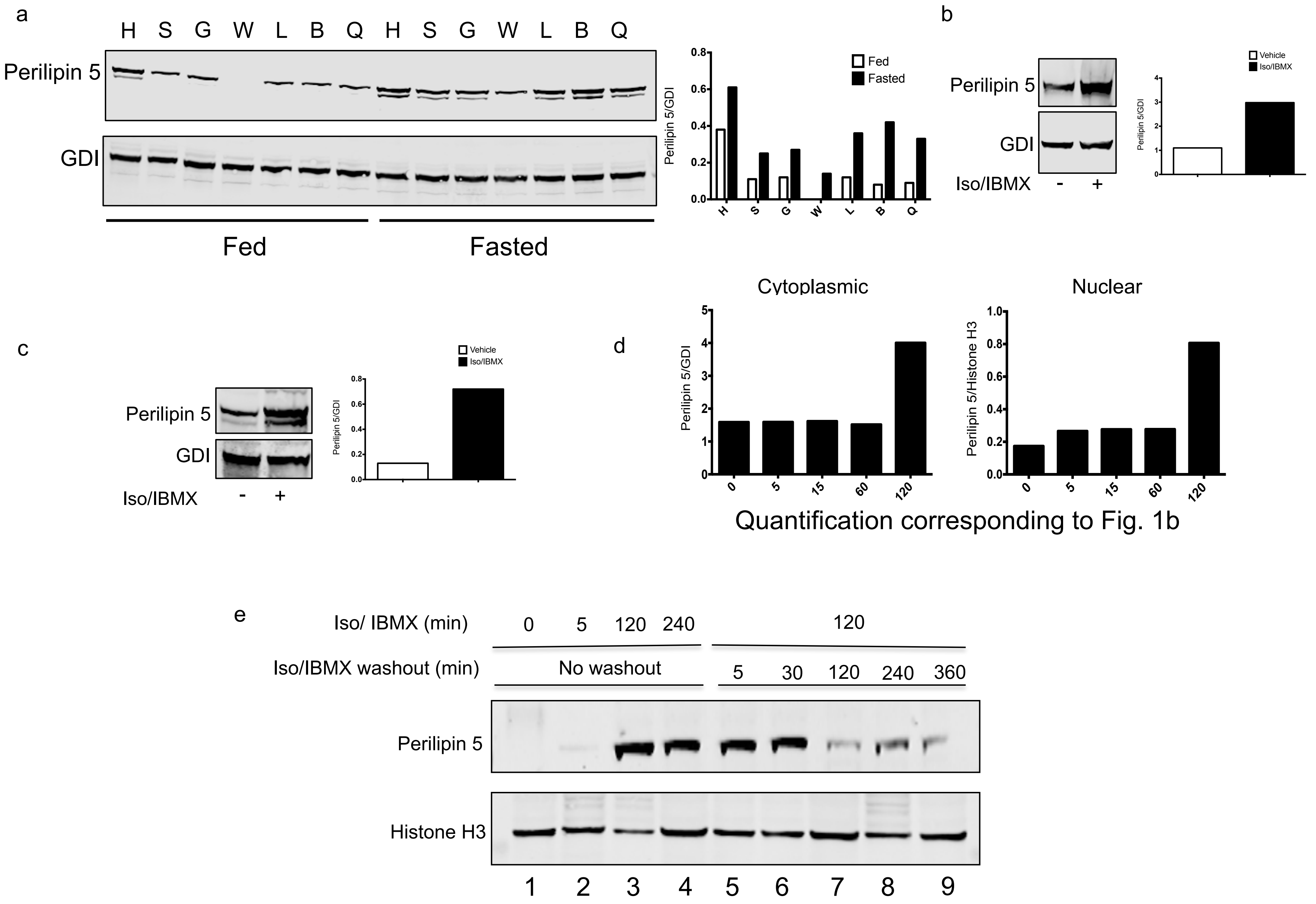

Supplementary Figure 2. Perilipin 5 protein expression and nuclear enrichment after catecholamine treatment.

a, Whole cell lysate prepared from heart (H), soleus (S), gastrocnemius (G), white adipose tissue (W) liver (L), brown adipose tissue (B) and quadriceps (Q), of wild type mice fed or fasted for 16 h (left panel) and corresponding quantification (right panel). **b and c,** Whole cell lysate prepared from C2C12 myotubes (b) or MLTC-1 (c) cells after treatment with Vehicle or Iso/IBMX and corresponding quantifications. **d,** Corresponding quantification for Fig. 1b. **e,** Lanes 1-4: C2C12 myotubes were treated with Iso/IBMX for 0 to 240 min as indicated and then nuclear fractions were prepared and immunoblotted for Perilipin 5 and histone H3. Lanes 5-9: C2C12 myotubes were treated with Iso/IBMX for 120 min and then the Iso/IBMX-containing medium was replaced with serum-free growth medium. At the indicated time points, nuclear fractions were prepared and immunoblotted as above.

Supplementary Figure 3

a

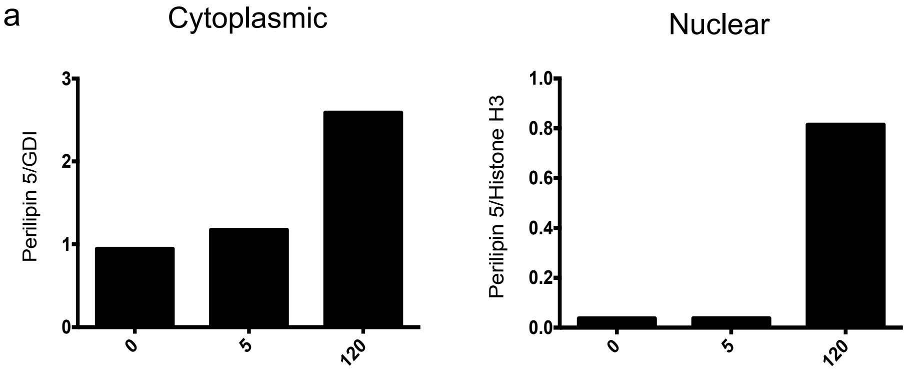

b

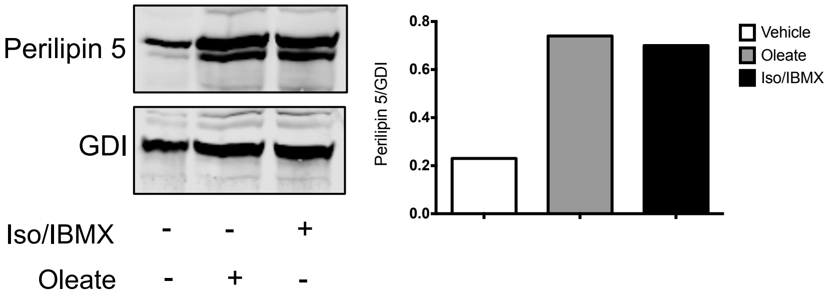

Quantification corresponding to Fig. 1c

c

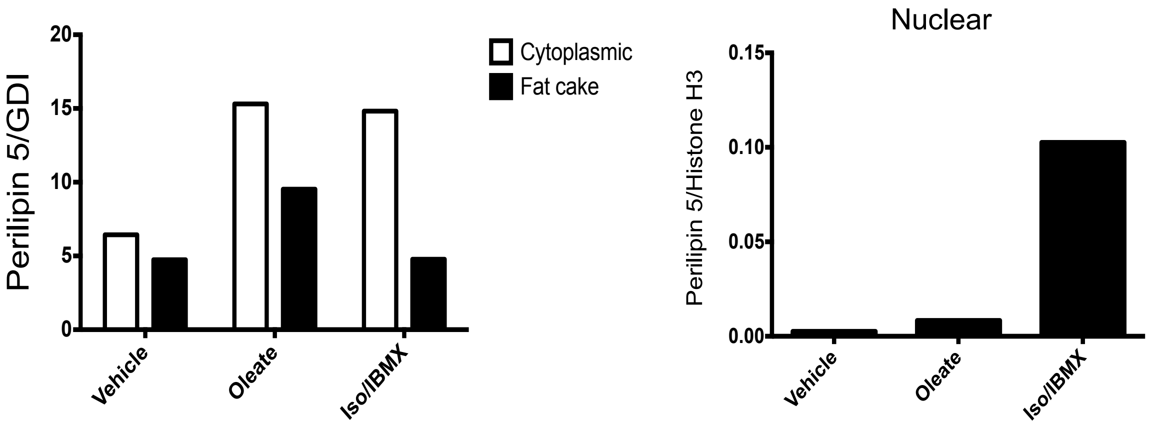

Quantification corresponding to Fig. 1d

Supplementary Figure 3. Perilipin 5 protein expression and nuclear enrichment after catecholamine treatment.

a, Western blot quantification corresponding to Fig. 1c. Data shown corresponds to the band intensity for Perilipin 5 divided by that of GDI as normalization for protein loading. **b,** Whole cell lysate from the MLTC-1 cells subjected to cell fractionation in Figure 1d with GDI-normalized Perilipin 5 quantification. **c,** Quantification of GDI-normalized Perilipin 5 for the cytoplasmic and fat cake fractions and histone H3-normalized Perilipin 5 for the nuclear fractions shown in Figure 1d.

Supplementary Figure 4

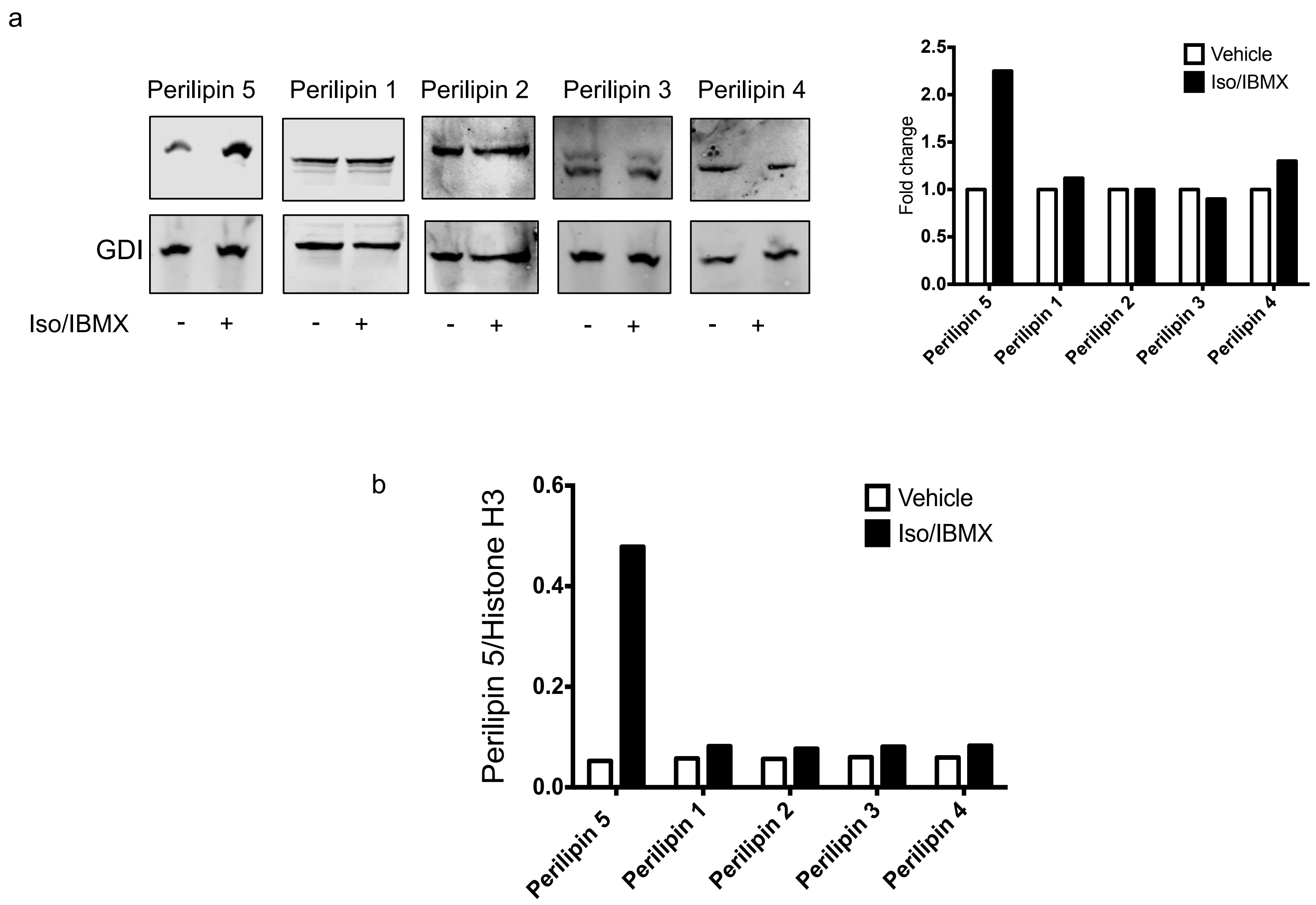

Quantification corresponding to Fig. 1e

Supplementary Figure 4. Perilipin 5 protein expression and nuclear enrichment.

a, Whole cell lysate from MLTC-1 cells treated with vehicle or Iso/IBMX and immunoblotted for Perilipins 1 through 5. The corresponding quantification indicates the fold-change in GDI-normalized Perilipin signal under Iso/IBMX versus vehicle treatment. **b,** Western blot quantification corresponding to Fig. 1e.

Supplementary Figure 5

a

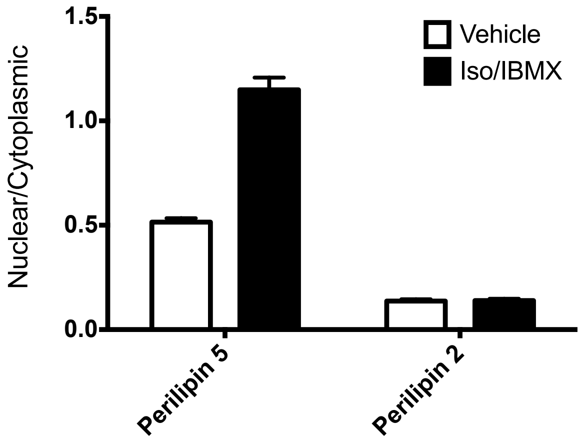

b

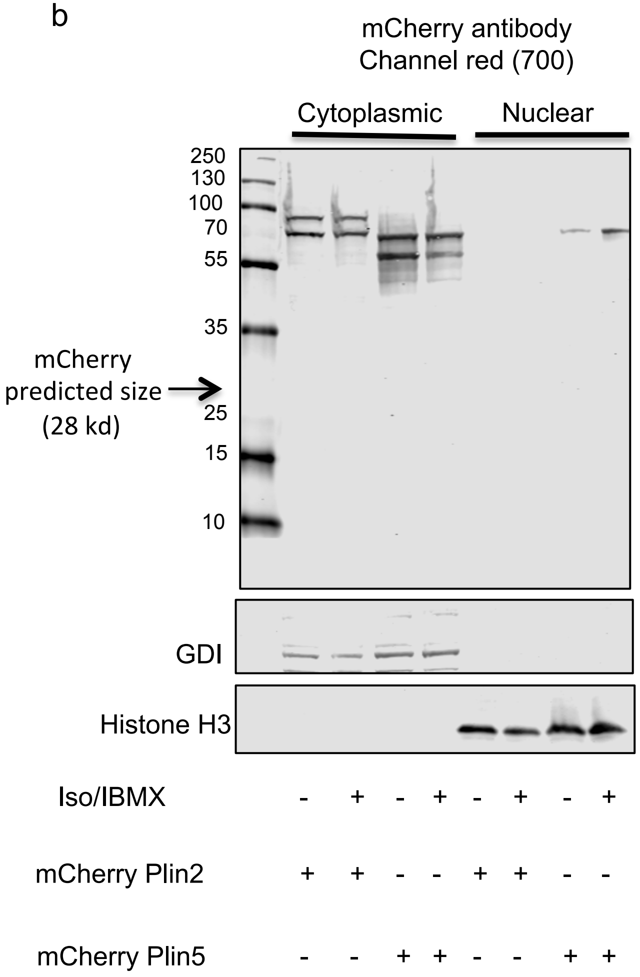

Supplementary Figure 5. mCherry-Perilipin 5 nuclear enrichment.

a, Nuclear/cytoplasmic ratio for quantification of Fig. 1f. Analysis was performed with Image J software in 25 individual cells. **b,** C2C12 myoblasts were transfected with mCherry-Perilipin 2 or mCherry-Perilipin 5, treated with vehicle or Iso/IBMX for 2 h, and then fractionated into nuclear and cytoplasmic fractions. The fractionated proteins were immunoblotted with antibody to mCherry.

Supplementary Figure 6

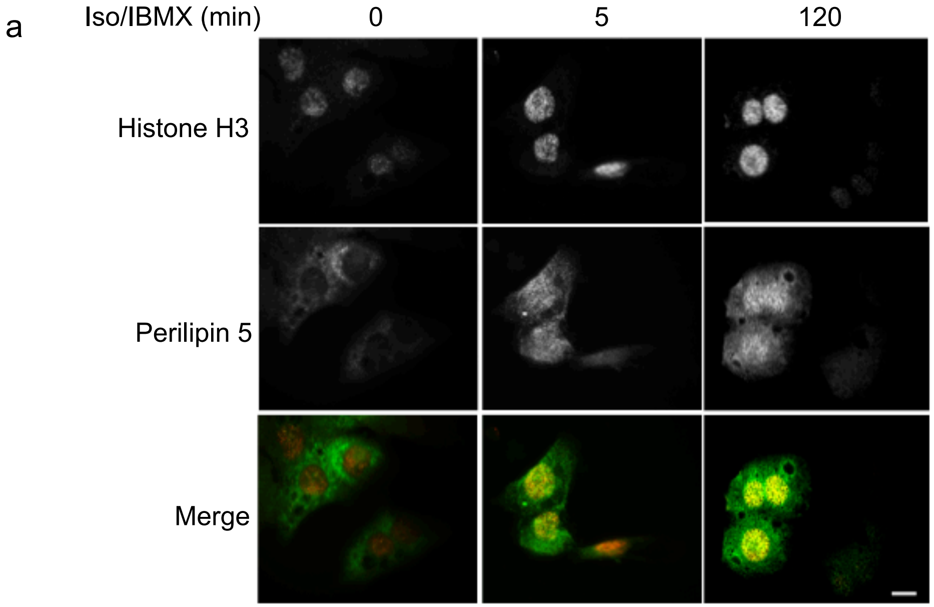

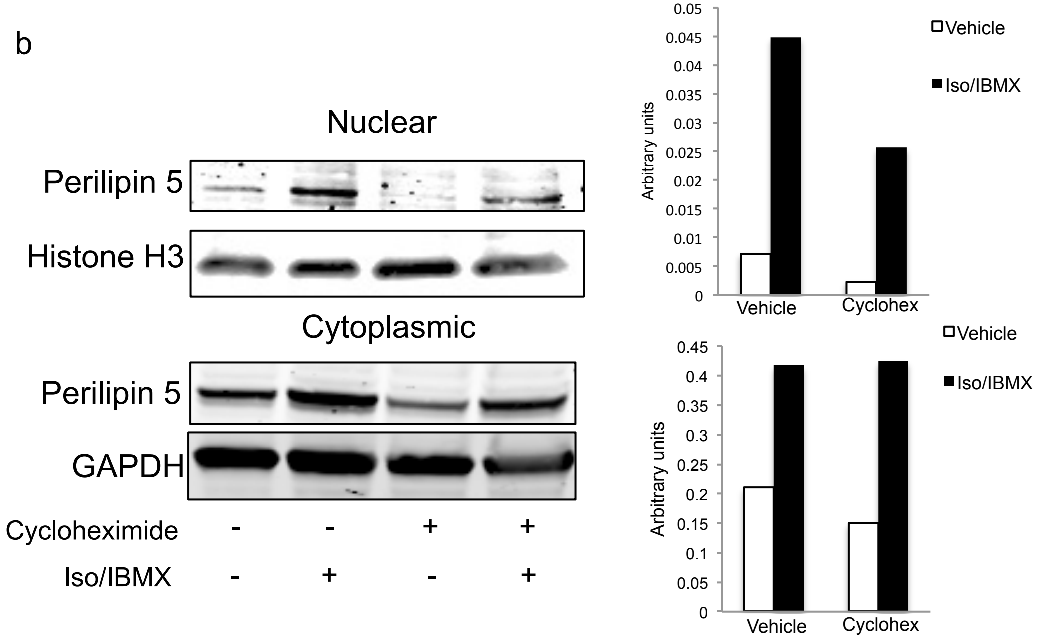

Supplementary Figure 6. Perilipin 5 nuclear enrichment.

a, Immunostaining of Perilipin-5 and nuclear marker histone H3 in MLTC-1 cells after Iso/IBMX treatment. Scale bar = 10μm. **b,** We treated C2C12 myotubes with 10 μg/ml of cycloheximide or vehicle for 2 h followed by treatment with vehicle or Iso/IBMX for 2 hours (along with cycloheximide or vehicle), we assessed Perilipin-5 in nuclear and cytoplasmic extracts by western blot (left panel) and western blot quantification and normalization (right panel).

Supplementary Figure 7

a

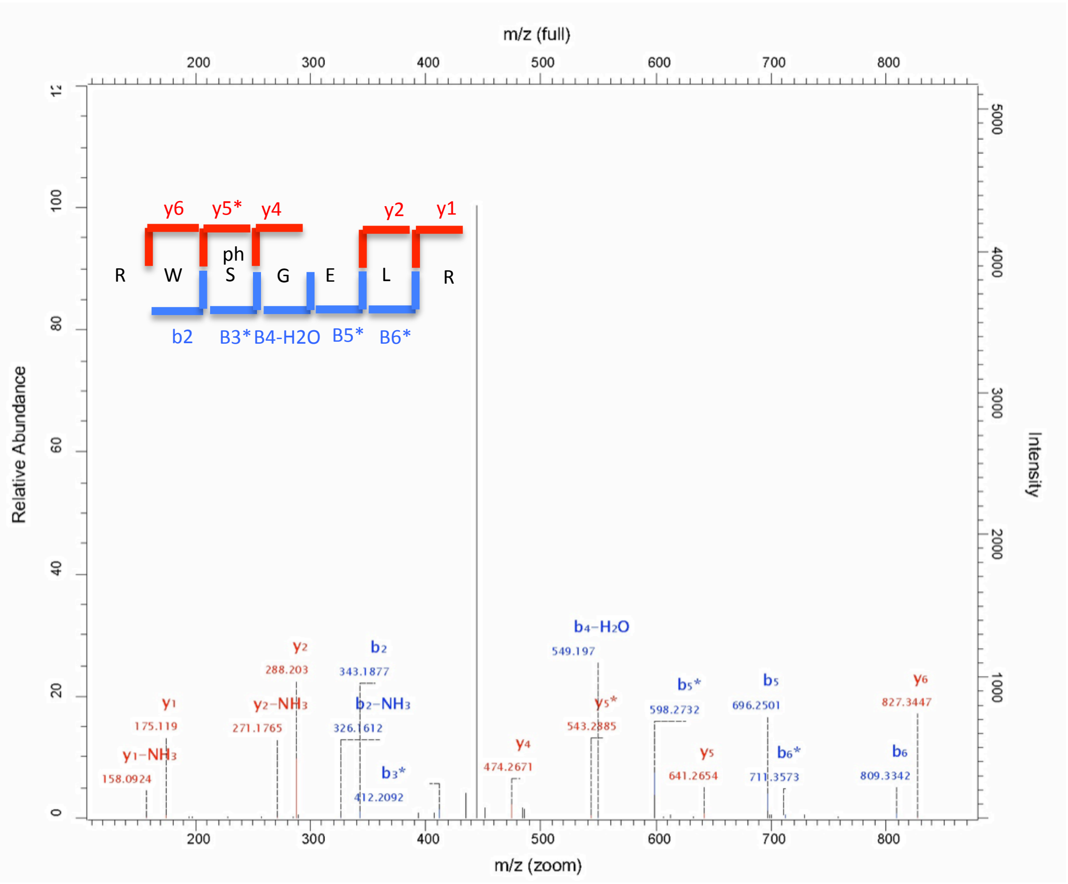

b

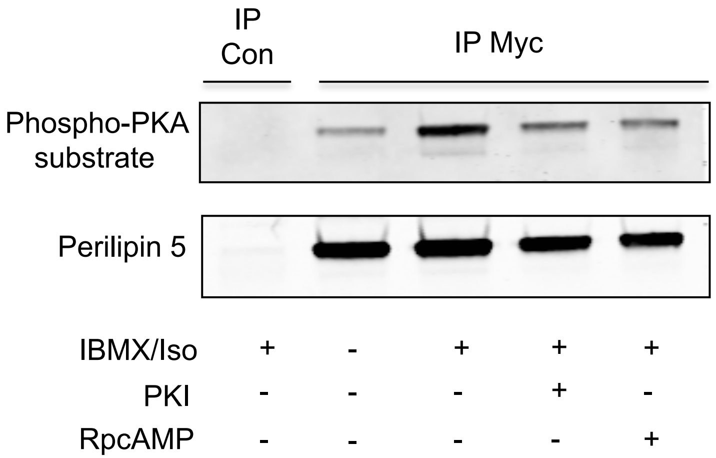

Supplementary Figure 7. Perilipin 5 S155 phosphorylation is PKA dependent

a, Using mass spectrometry we identified serine 155 phosphorylation of Perilipin 5 under Iso/IBMX treatment. The MS/MS spectrum shows the product ions scan of the double charged phosphorylated peptide RWSGELR. **b,** We treated OP9 Myc-Perilipin 5 adipocytes with Iso/IBMX and PKA inhibitors or vehicle (pre-incubation for 1 h with PKI 10 µM or RpcAMP 50 µM followed by Iso/IBMX for 2 h). For each condition we performed Perilipin-5 immunoprecipitation followed by immunoblotting with a phospho-PKA substrate antibody.

Supplementary Figure 8

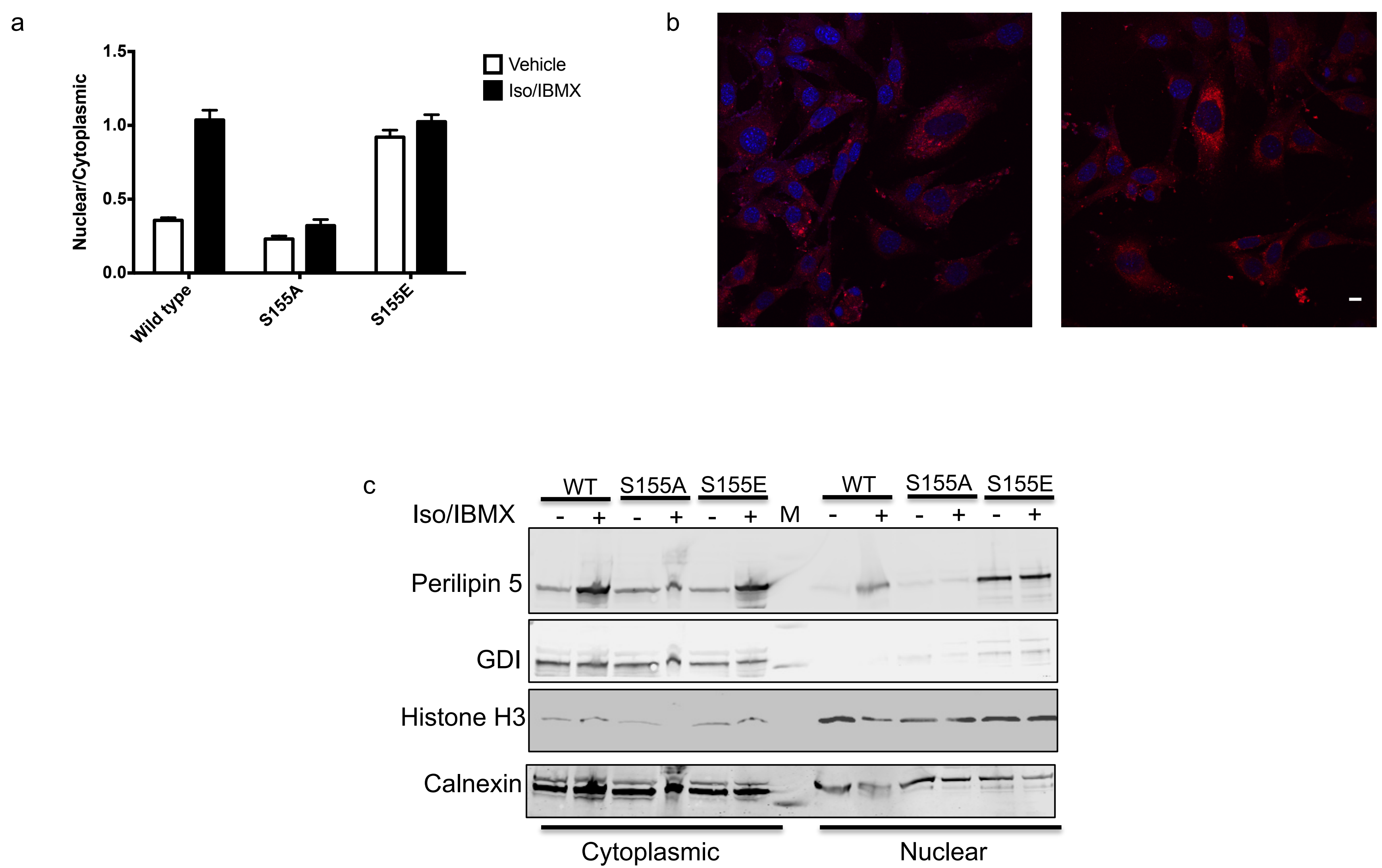

Supplementary Figure 8. Perilipin 5 S155 phosphorylation and nuclear enrichment

a, Nuclear/cytoplasmic ratio for quantification of nuclear enrichment in Fig. 1h. Analysis was performed with Image J software in 25 individual cells. **b,** Wild type Perilipin 2 was transfected into C2C12 myoblasts followed by treatment by vehicle or Iso/IBMX for 2 hours and then immunostaining for Perilipin 2 and nuclear staining with DAPI. Scale bar = 10μm. **c,** Nuclear and cytoplasmic fractions of C2C12 myoblasts transfected with wild type Perilipin 5, S155A or S155E followed by treatment with vehicle or Iso/IBMX for 2 hours.

Supplementary Figure 9

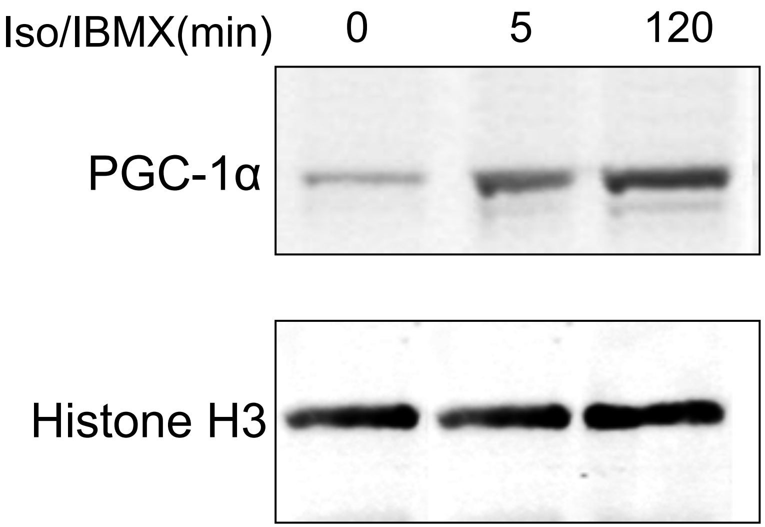

Supplementary Figure 9. PGC1-α protein expression is increased with Iso/IBMX treatment.

Western blot for PGC-1α and histone H3 in nuclear fractions from C2C12 myotubes treated with Iso/ IBMX for 5 and 120 min.

Supplementary Figure 10

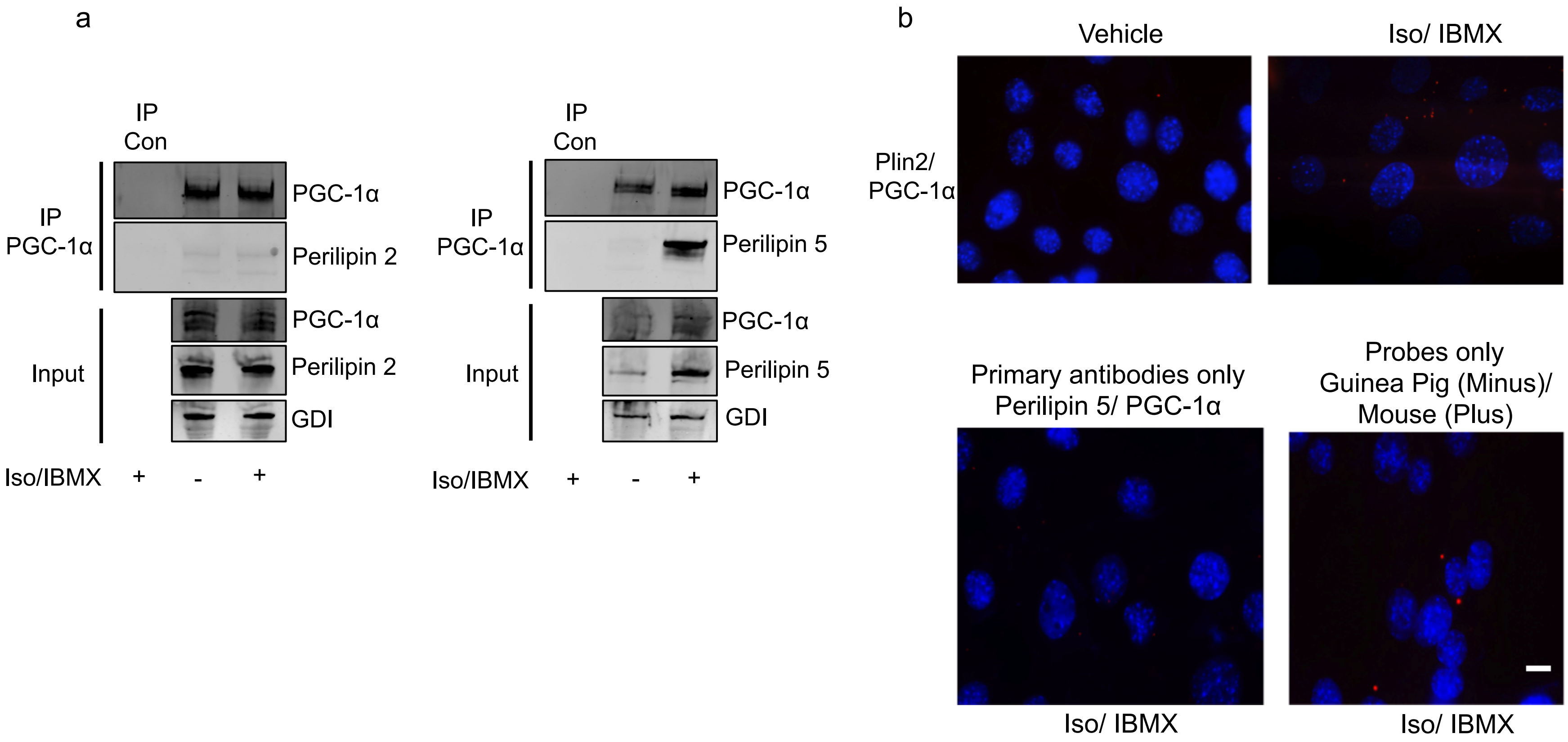

Supplementary Figure 10. PGC1-α and Perilipin 2 do not form catecholamine-stimulated complexes.

a, IP of PGC-1α from whole cell lysate of C2C12 myoblasts transfected with PGC-1α and Perilipin 2 (left panel) or PGC-1α and Perilipin 5 (right panel) after treatment with vehicle or Iso/IBMX for 2 h. **b,** Top Panels: Proximity ligation assays (PLA) reveal no significant nuclear signals to indicate interaction between Perilipin 2 and PGC-1α under either vehicle or Iso/IBMX treatment. Bottom Panels: As negative controls we performed PLA using only primary antibodies for Perilipin 5 and PGC-1α with no secondary antibodies or the guinea pig and mouse secondary antibody probes without primary antibodies. Scale bar = 10μm.

Supplementary Figure 11

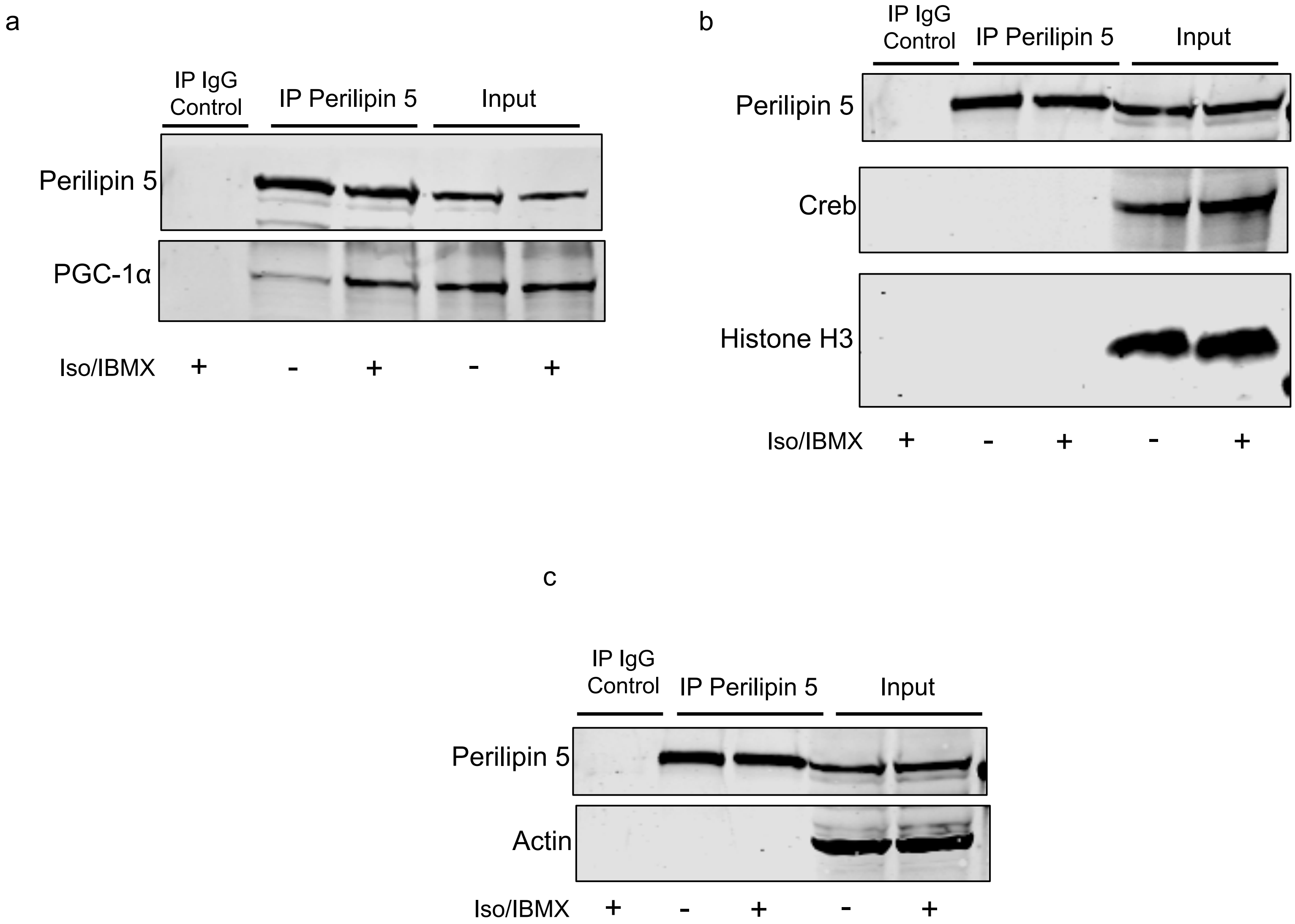

Supplementary Figure 11. Perilipin 5 does not form complexes with Creb, Histone H3 or Actin.

IP of Perilipin 5 from whole cell lysate of C2C12 myotubes treated with Vehicle or Iso/IBMX. IP and input samples were loaded for three separate western blots in order to immunoblot with several antibodies as follows **a,** Perilipin 5 and PGC1-α. **b,** Perilipin 5, Creb and Histone H3 and **c,** Perilipin 5 and actin.

Supplementary Figure 12

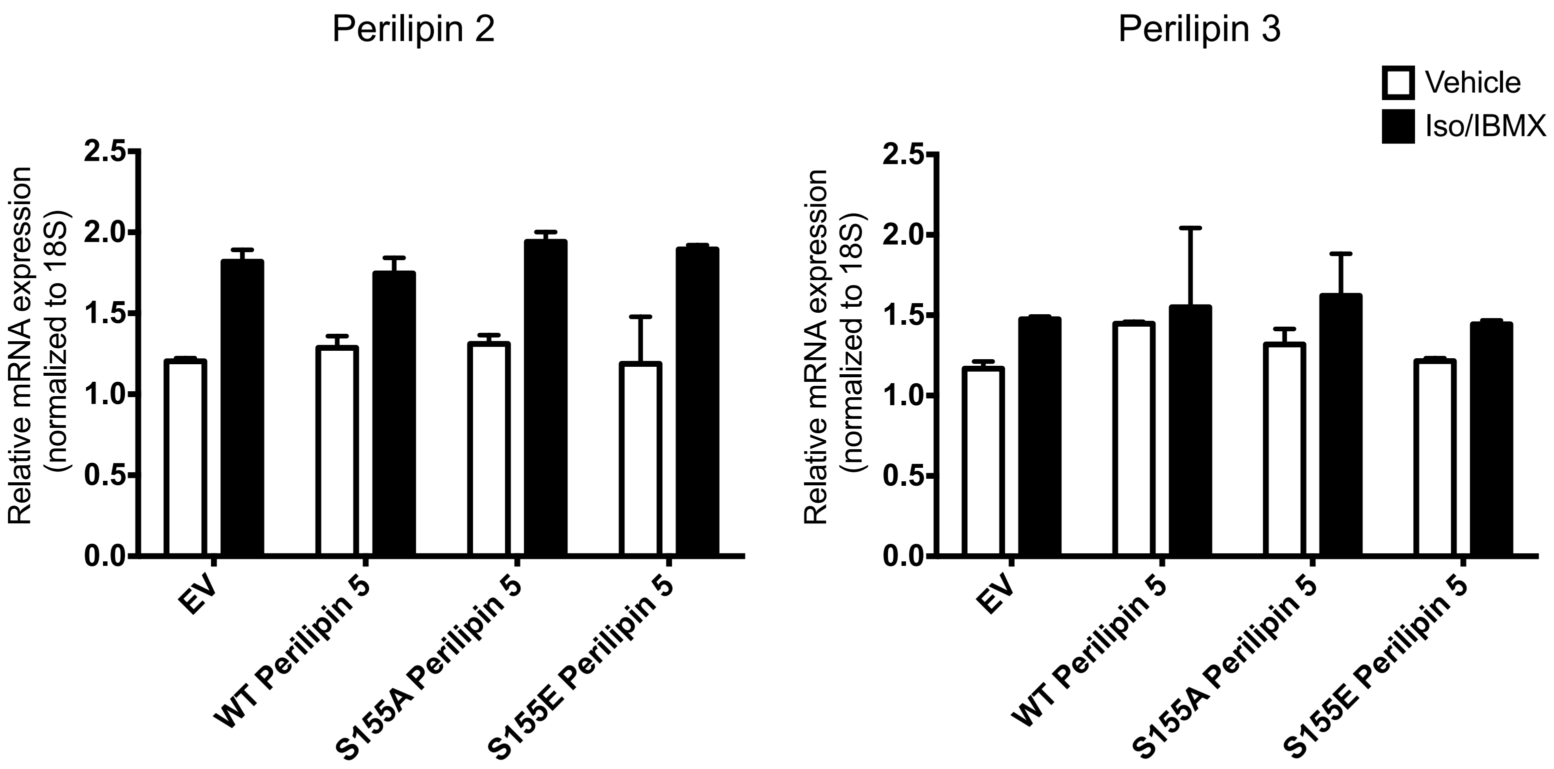

Supplementary Figure 12. Perilipin 5 overexpression does not change Perilipin 2 or Perilipin 3 gene expression.

Perilipin 2 and Perilipin 3 gene expression measured by qPCR in the experiment shown in Fig. 3a. qPCR was used detect expression of indicated genes in C2C12 myoblasts individually transfected the following plasmids: empty vector (EV) wild type myc-Perilipin 5, S155A Perilipin 5, and S155E Perilipin 5, followed by treatment with vehicle or Iso/IBMX for 2 h. Values are mean ± s.e.m. n=3/group.

* $p < 0.05$ compared to Vehicle and, $^{\#}$ $p < 0.05$ compared to EV and $^{\&}$ $p<0.05$ compared to wild type using ANOVA followed by Tukey post-test.

Supplementary Figure 13

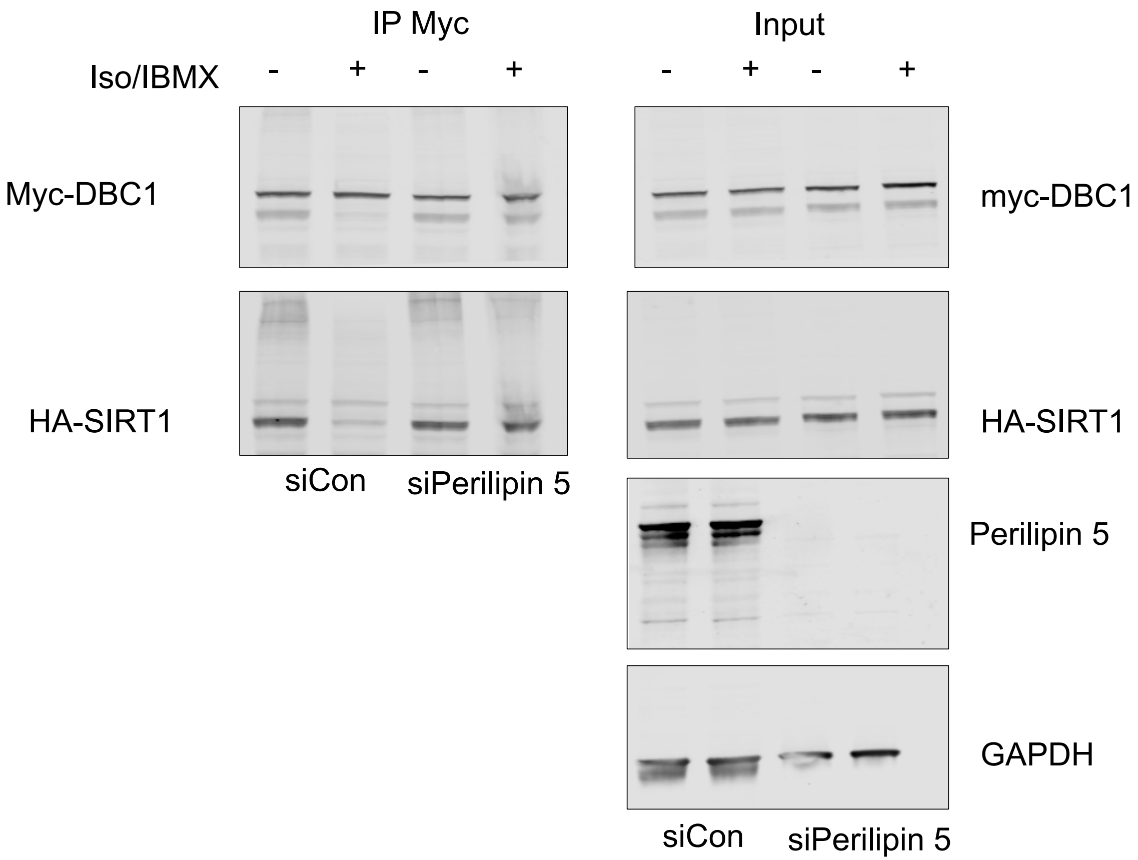

Supplementary Figure 13. Regulation of SIRT1/DBC1 complex by Perilipin 5. Myc-DBC and HA-SIRT1 expression plasmids were co-transfected into MLTC-1 cells with either siControl or siPerilipin 5 and then treated with vehicle or Iso/IBMX for 30 min. Myc-DBC1/HA-SIRT1 complexes were then immunoprecipitated using myc antibody and the immunoprecitated blotted for with myc and HA antibodies. Inputs were immmunoblotted for myc, HA, Perilipin 5 and GAPDH.

Supplementary Figure 14

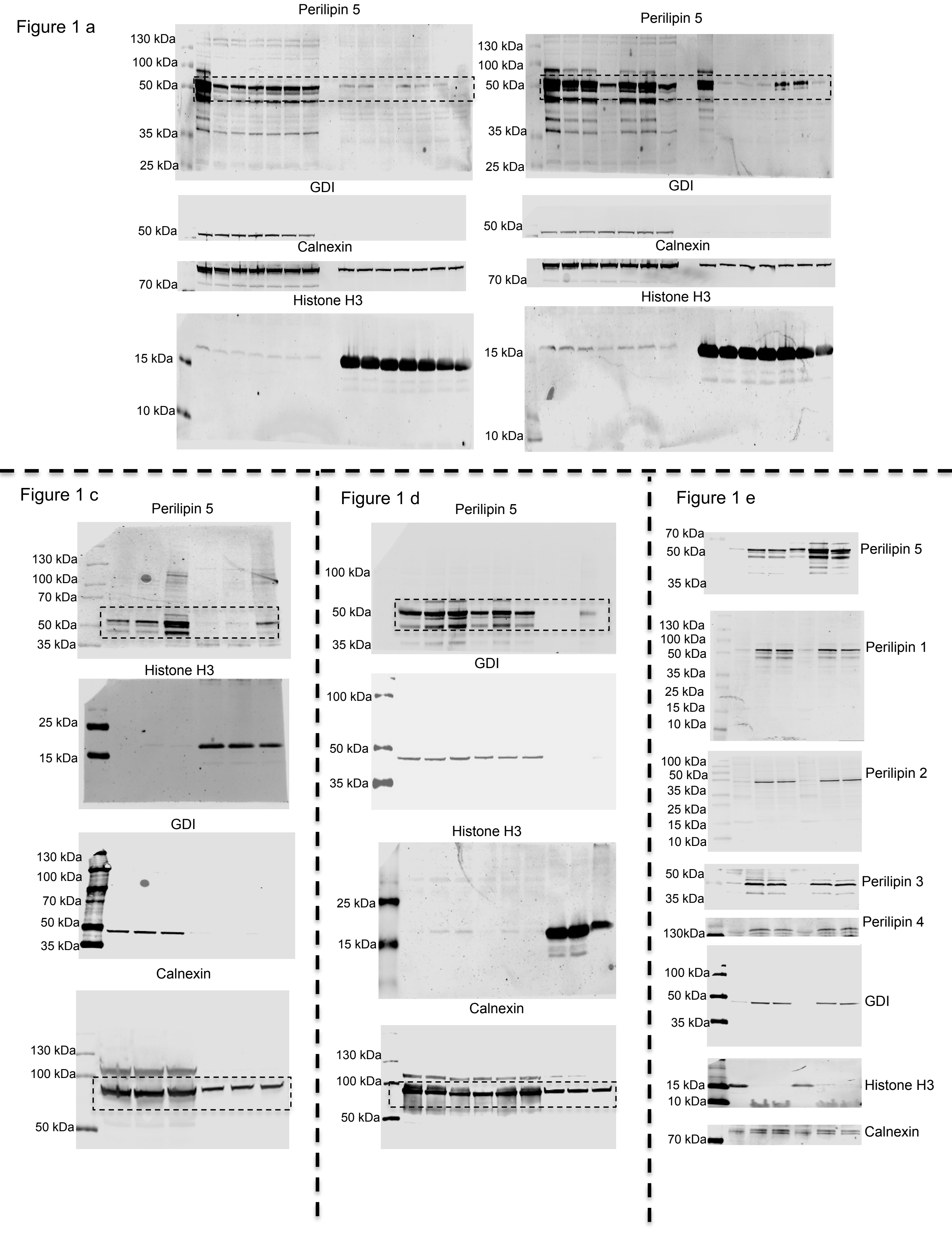

Figure 2 a

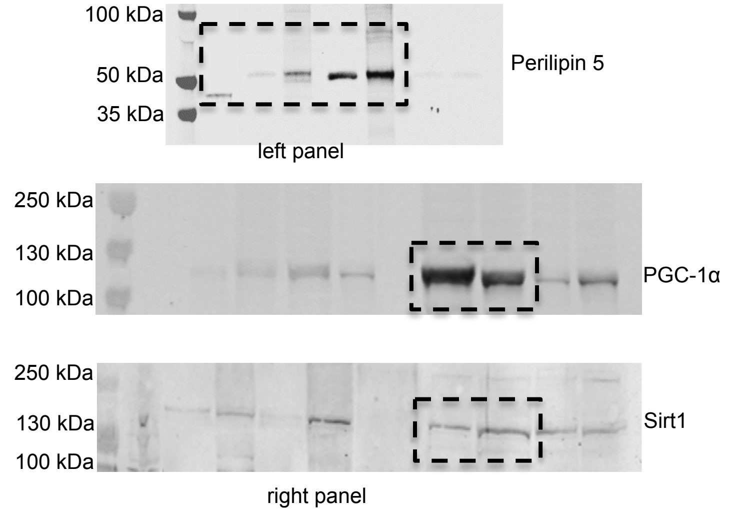

Figure 2 d

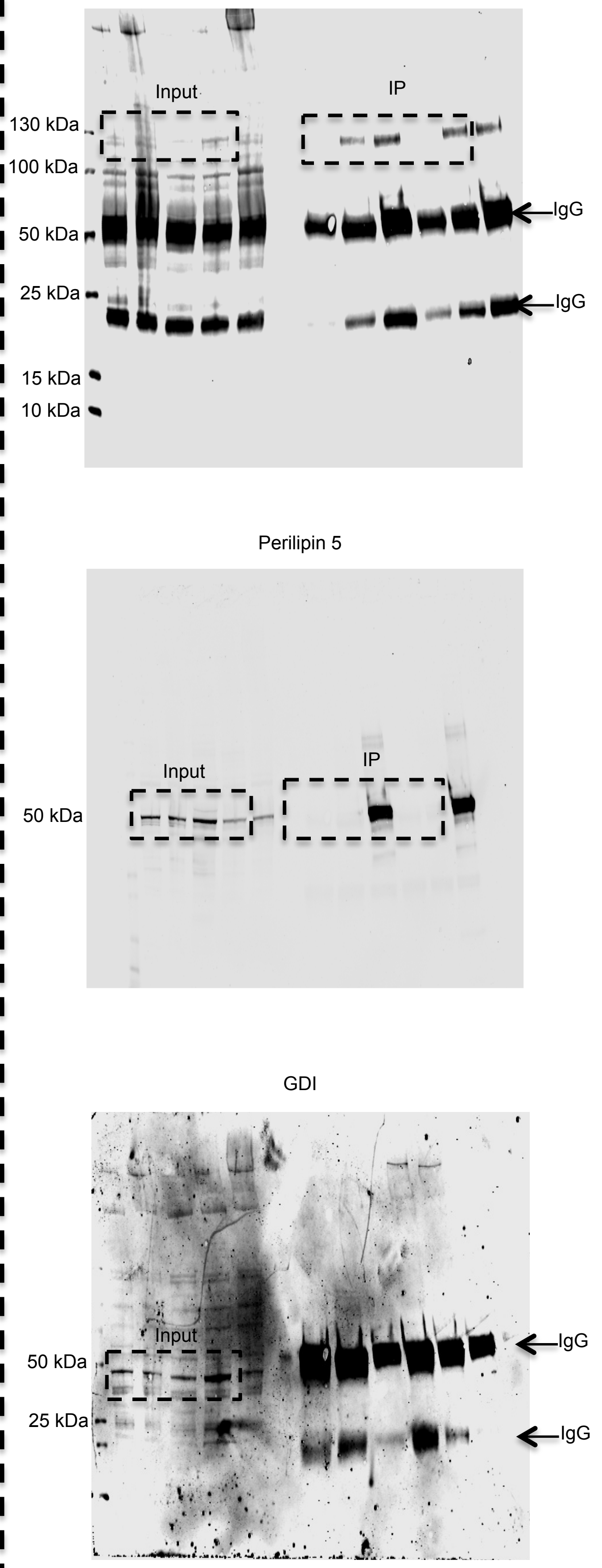

Figure 2 e

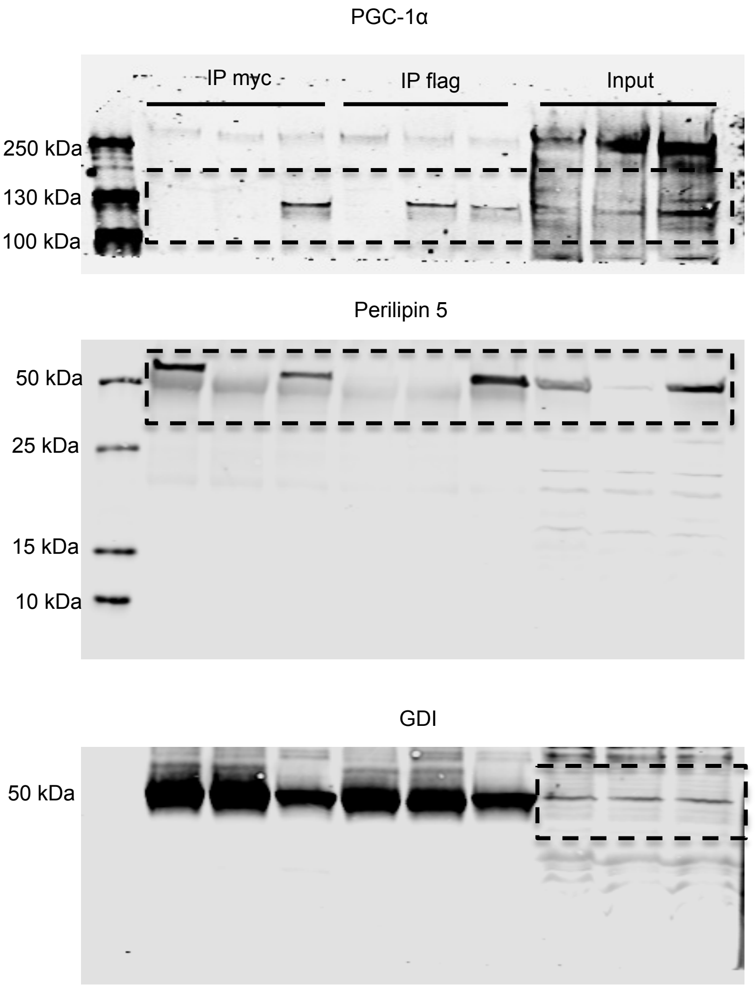

Figure 2 h

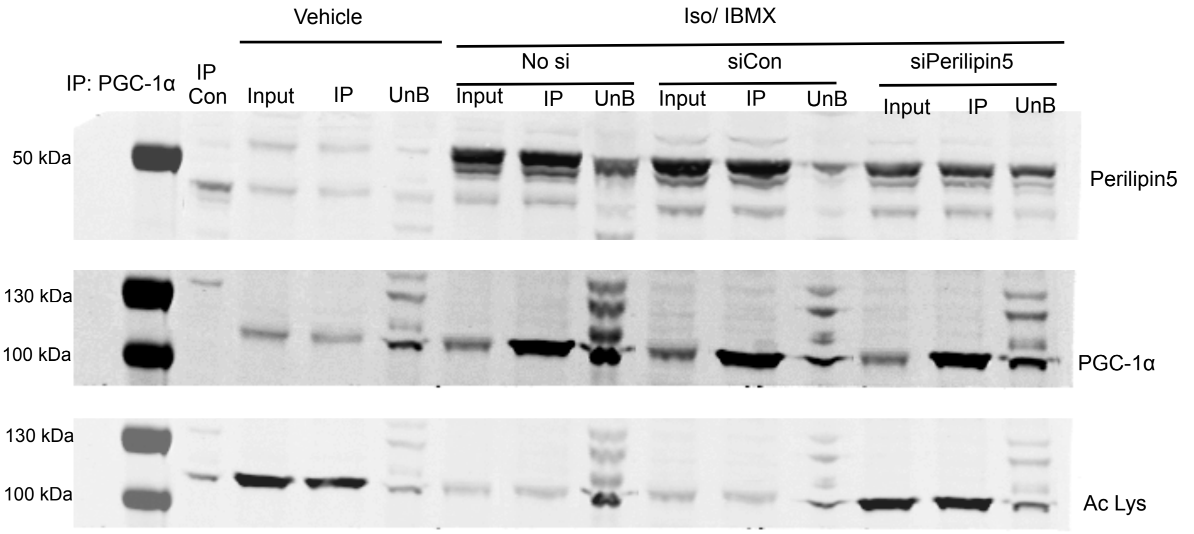

Figure 2 g

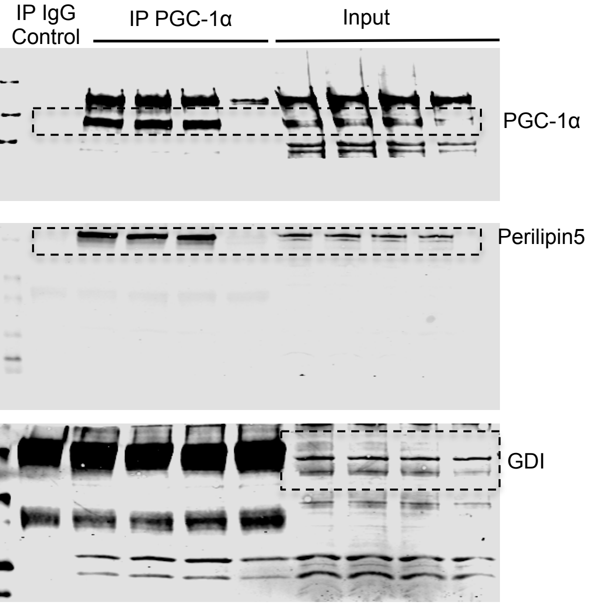

Figure 6 b

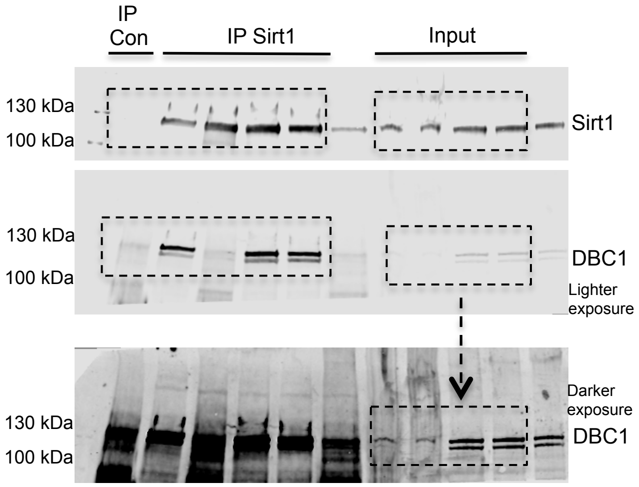

Supplementary Figure 10 left panel

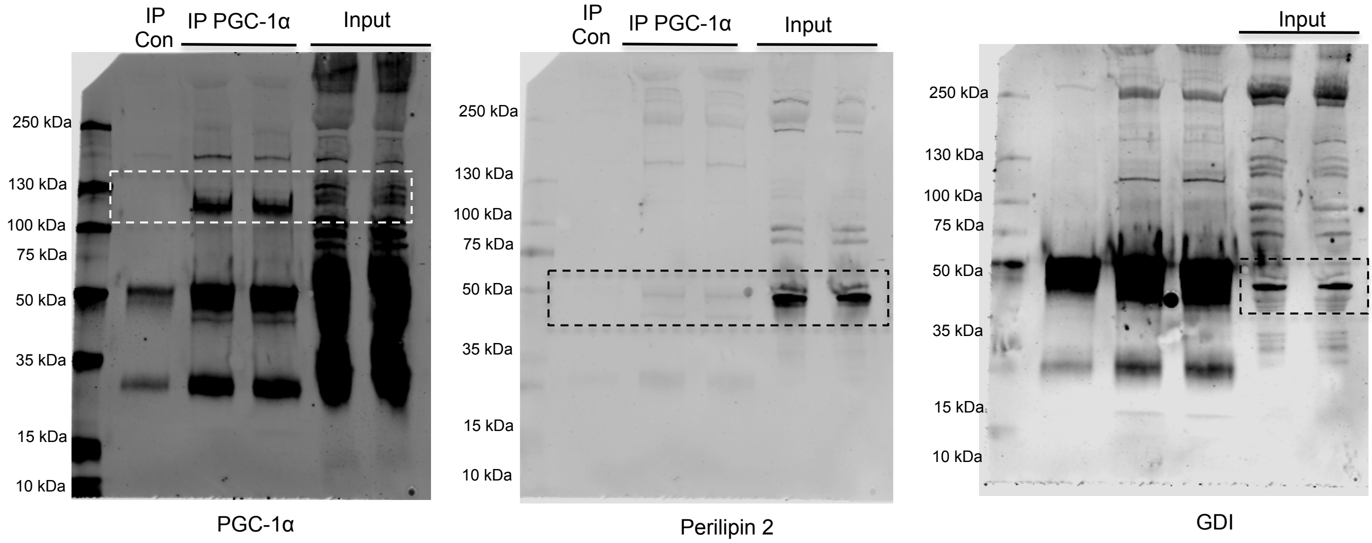

Supplementary Figure 10 center panel

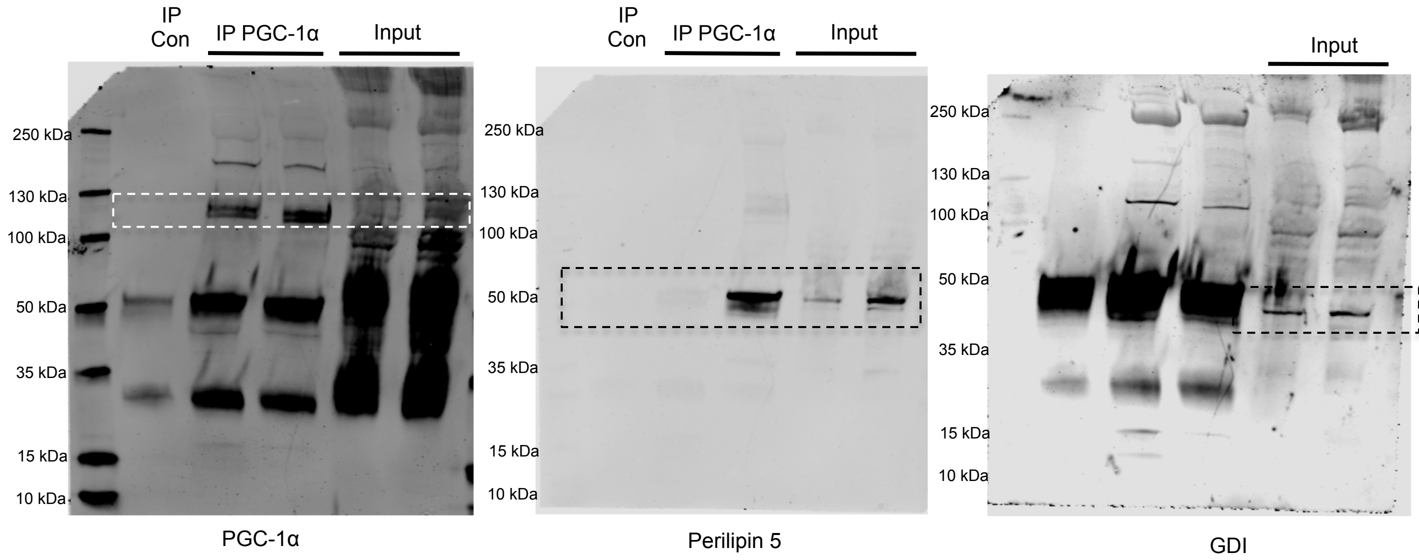

Supplementary Figure 11

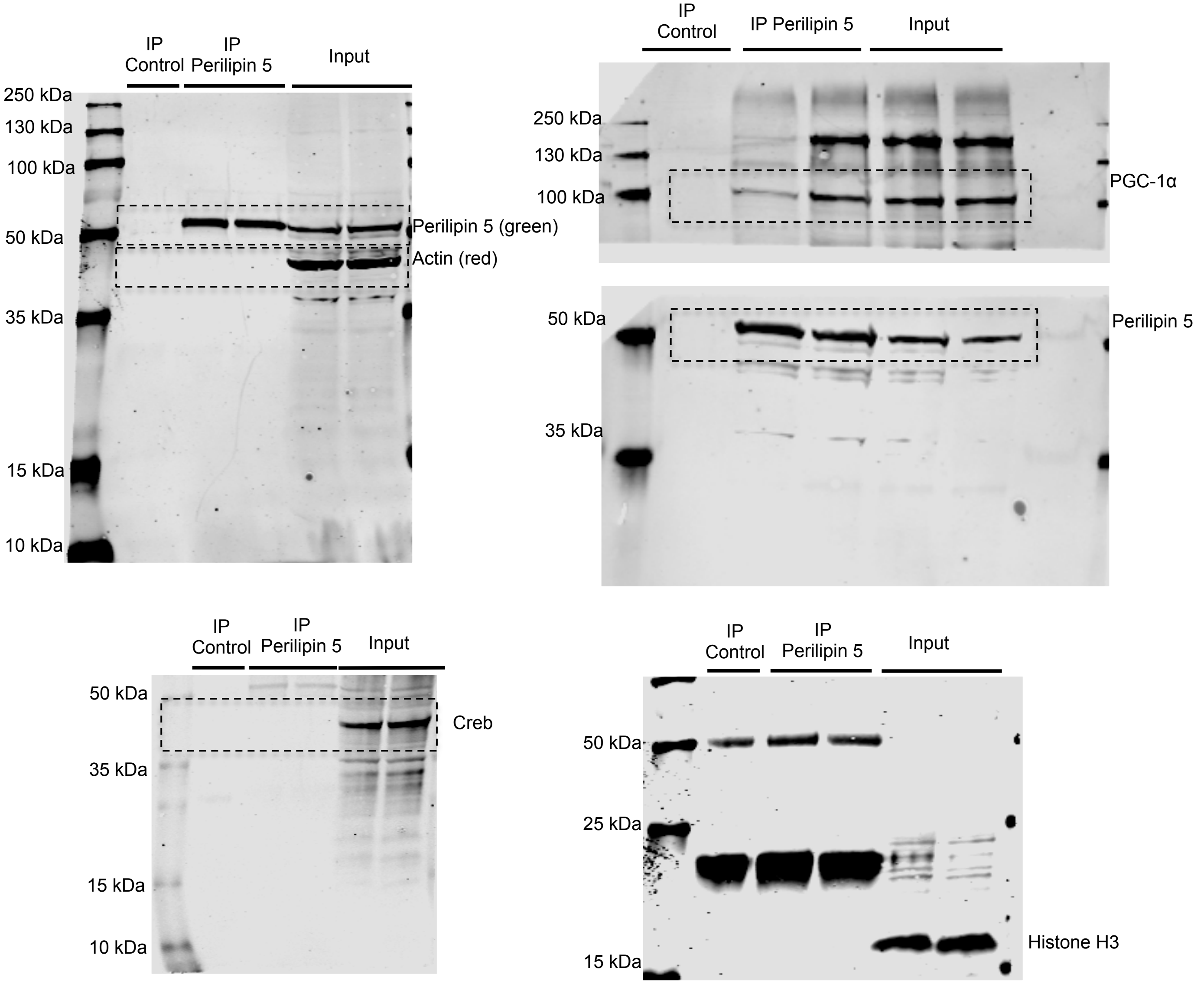

Supplementary Figure 14. Most important un-cropped western blots.

Within each figure the individual blots correspond to the same membrane cut into panels according to molecular weight range. Each panel was then probed for the proteins indicated in the figure. In some cases (as indicated on Supplementary Figures 1c, 1d, and 1e), equal amounts of cellular protein from the same sample were resolved on separate gels and immunoblotted for the proteins indicated. For Figure 1e, the panels for Perilipin 5, GDI, and Histone H3, were cut from the same immunoblot; whereas, panels probed for Perilipins 1-4 and calnexin represent individual western blots.

Supplementary Table 1. Primers used for qPCR

Plin5	Forward: CCATCTCGCCTATGAACACTCTT<br>Reverse: CAGCTGGGCCAGCATCTC
Sirt1	Forward: TGTGAAGTTACTGCAGGAGTGTAAA<br>Reverse: GCATAGATACCGTCTCTTGATCTGAA
Tfam	Forward: CCAAAAAGACCTCGTTCAGC<br>Reverse: ATGTCTCCGGATCGTTTCAC
Nampt	Forward: CCGCCACAGTATCTGTTCCTT<br>Reverse: AGTGGCCACAAATTCCAGAGA
Ppargc1a	Forward: AACCACACCCACAGGATCAGA<br>Reverse: TCTTCGCTTTATTGCTCCATGA
Esrraγ	Forward: ACTTGGCTGACCGAGAGTTG<br>Reverse: GCCAGGGACAGTGTGGAGAA
Pdk4	Forward: AAGCAAAACACAAACACGAGTA<br>Reverse: CCCGGGTCATCCAACCA
Ucp3	Forward: CCTACGACATCATCAAGGAGAAGTT<br>Reverse: TCCAAAGGCAGAGACAAAGTGA
Cpt1	Forward: CACCAACGGGCTCATCTTCTA<br>Reverse: CAAAATGACCTAGCCTTCTATCGAA
Cyclophilin	Forward: TGGAGAGCACCAAGACAGACA<br>Reverse: TGCCGGAGTCGACAATGAT
Erssα	Forward: CTCAGCTCTCTACCCAAACGC<br>Reverse: CCGCTTGGTGATCTCACACTC
Ucp1	Forward: CCCTGGCAAAAACAGAAGGA<br>Reverse: AGCTGATTTGCCTCTGAATGC
Elovl3	Forward: GCCAAACTGAAGCATCCTAATCTT<br>Reverse: CCCAGAACCATCTGCAGAATC
18S	Forward: GTAACCCGTTGAACCCCATT<br>Reverse: CCATCCAATCGGTAGAGCG

Supplementary Table 2. Primers used for ChIP

**PGC-1a promoter**	P1 Forward: CAGGAGTTTGTGCAGCAAGCT<br>P1 Reverse: ATTAAAAAGTAGGCTGGGCTGTCA
	P2 Forward: AGGTGCCTTCAGTTCACTCTCAGT<br>P2 Reverse: AGCTTTTTCAACTCCAATCCACTCT
	P3 Forward: AGCTGATCTGAGCAGAGCAG<br>P3 Reverse: CTCAAGCTCAGTTTGGGACT
	P4 Forward: GATGCTTGAAGCCTCCCAAA<br>P4 Reverse: TCTGCCTCAGTGAAGTAACGCTTA

Supplementary Table 3. Primers used for site-directed mutagenesis

Plin5S155A	Forward: GGGCCGGCGTTGGGCTGGGGAGCTGAGG<br>Reverse: CCTCAGCTCCCCAGCCCAACGCCGGCCC
Plin5S155D	Forward: GGGCCGGCGTTGGGATGGGGAGCTGAGG<br>Reverse: CCTCAGCTCCCCATCCCAACGCCGGCCC
Plin5S155E	Forward: GGGGCCGGCGTTGGGAGGGGGAGCTGAGGCG<br>Reverse: CGCCTCAGCTCCCCCTCCCAACGCCGGCCCC

Supplementary Table 4. Primers used for mitochondrial DNA.

Cox 2	Forward: ATAACCGAGTCGTTCTGCCAAT<br>Reverse: TTTCAGAGCATTGGCCATAGAA
Rsp18	Forward: TGTGTTAGGGGACTGGTGGACA<br>Reverse: CATCACCCACTTACCCCCAAAA
